# Robust unsupervised deconvolution of linear motifs characterizes 68 protein modifications at proteome scale

**DOI:** 10.1038/s41598-021-01971-3

**Published:** 2021-11-18

**Authors:** Theodore G. Smith, Anuli C. Uzozie, Siyuan Chen, Philipp F. Lange

**Affiliations:** 1 Department of Pathology, University of British Columbia, Vancouver, BC Canada; 2 Department of Computer Science, University of British Columbia, Vancouver, BC Canada; 3 Michael Cuccione Childhood Cancer Research Program, BC Children’s Hospital Research Institute, 950 West 28th Avenue, Vancouver, BC V5Z 4H4 Canada

**Keywords:** Proteome informatics, Software, Statistical methods, Systems biology

## Abstract

The local sequence context is the most fundamental feature determining the post-translational modification (PTM) of proteins. Recent technological improvements allow for the detection of new and less prevalent modifications. We found that established state-of-the-art algorithms for the detection of PTM motifs in complex datasets failed to keep up with this technological development and are no longer robust. To overcome this limitation, we developed RoLiM, a new linear motif deconvolution algorithm and webserver, that enables robust and unbiased identification of local amino acid sequence determinants in complex biological systems demonstrated here by the analysis of 68 modifications found across 30 tissues in the human draft proteome map. Furthermore, RoLiM analysis of a large-scale phosphorylation dataset comprising 30 kinase inhibitors of 10 protein kinases in the EGF signalling pathway identified prospective substrate motifs for PI3K and EGFR.

## Introduction

The state of the proteome is shaped by post-translational modification affecting the majority of proteins in one form or another^1,2^. Mass spectrometry allows for the de-novo detection of unknown modifications and technological advancements in enrichment procedures, top-down approaches and instrument sensitivity, have led to a steep increase in the modifications known to occur in vivo^3,4^. In addition to phosphorylation, the modification enabling rapid protein state changes underlying most cellular signaling cascades, more and more modifications are recognized for their wide-spread occurrence, functional effects^5–7^, and implications in disease pathology^8–10^. With the increase in identification of more and lower prevalence modifications, their classification and functional interpretation have become increasingly important, yet remain challenging. While many factors, including localization, folding and presence of interaction interfaces or auxiliary proteins, influence if a protein is a suitable substrate for an enzyme, the most fundamental requirement is the ability of the sequence surrounding the modified residue to interact with and fit into the active site of the enzyme. Protein modifying enzymes therefore leave a telltale signature that has been characterized for many enzymes in great detail using biochemical and cell biological approaches^11,12^. While direct experimental characterization of these short, usually 5–15 amino acid long linear motifs, provides the most direct link between an enzyme and its sequence requirements, it remains time consuming. Its low throughput is also outpaced by the observation of new modifications^13–15^. Computational approaches complement experimental characterization in two main ways. Firstly, they enable classification of enzymatic activities in biological systems based on known characteristics of experimentally studied enzymes. Recent examples of these primarily machine learning-driven algorithms include the identification of kinase activities in complex samples^16–18^. Secondly, algorithms based on calculating the enrichment of residues and residue patterns over normal background frequencies enable the deconvolution of experimentally-determined preferred substrate sequences into multiple independent linear motifs^19–23^. They further enable the unbiased characterization of modified sequences without prior knowledge and experimental characterization of the underlying enzyme^23,24^. The gold standard algorithm for linear motif deconvolution, Motif-X^20,23^ has been in use for over 15 years and is no longer available. It has been re-implemented without major modifications^21,25^ and continues to be widely used and cited > 100 times per year. Here we hypothesized that current data from proteome-wide, pan-enzyme or pan-modification studies may pose challenges that were not present when the algorithm and re-implementations were initially conceived and validated. We found previously undescribed limitations in the robustness and accuracy of motif detection using current re-implementations of Motif-X. rmotif-X showed strong preference for detecting patterns enriched on the N terminal side of the modified central residue while motif deconvolution in Momo was very sensitive to the order of sequence arrangement in the input data.

## Results and discussion

**Figure 1:**
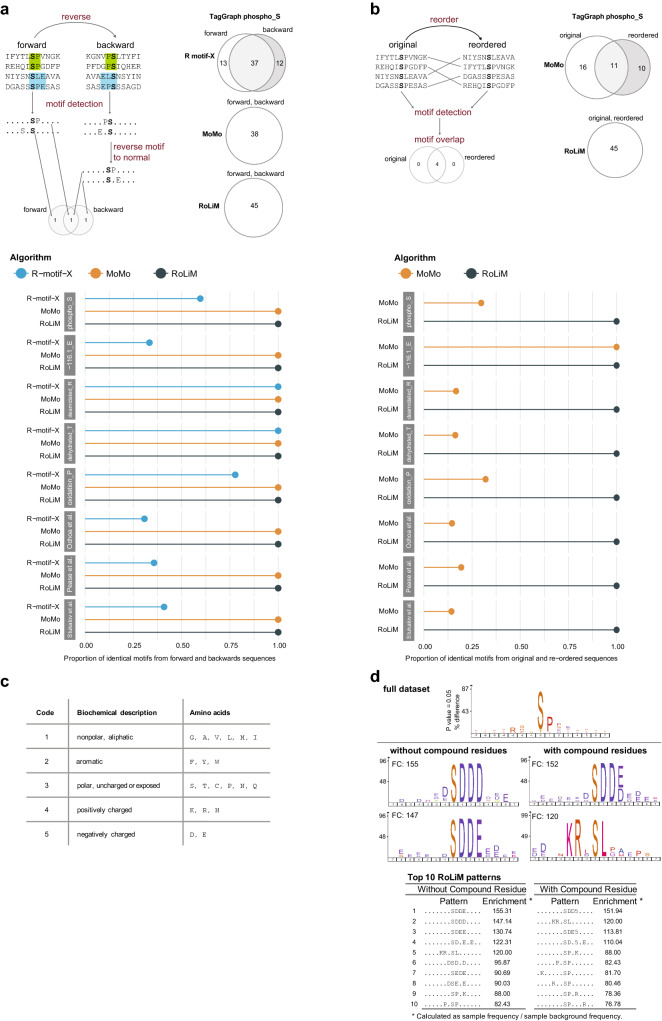
Positional bias and performance comparison between algorithms. (**a**) Overlap between motifs identified by rmotif-X, MoMo, and RoLiM in original and reverse sequence datasets. (**b**) Overlap between motifs identified by rmotif-X, MoMo, and RoLiM in original and reordered sequence datasets. The proportion of overlap shown in the lollipop graph was calculated as the number of the same motifs found in both forward and reverse foregrounds/the sum of all unique motifs found in both foregrounds. See online Methods for details. (**c**) Compound residue classification implemented in RoLiM. (**d**) Top ten enriched phopsho-serine motifs identified by RoLiM in the dataset from Zadora et al. with and without compound residues enabled.

The largest pan-modification dataset to date covers 73 modifications. It was generated based on the human proteome draft^26^ raw data using the TagGraph unrestricted search strategy^14^. When we analyzed the local sequence context of protein modifications identified in the TagGraph dataset we noticed that residues found as enriched by rmotif-X were not equally distributed around the modified central residue. Patterns identified by rmotif-X for 41 of 73 modifications showed more enriched residues on the N-terminal side of the modified residue. We wondered if this was a true characteristic of the modifications or a bias introduced by the motif extraction algorithm. We hypothesized that a biased algorithm should identify different enriched motifs if presented with inverted (C to N terminus) foreground and background sequences. Since the original Motif-X implementation has not been available for over a year we could not test this hypothesis for the original algorithm but could only evaluate the two re-implementations rmotif-X and MoMo. Using the rmotif-X implementation^20^, we indeed found strong evidence of positional bias in 8 of 9 tested datasets^27–29^ (Fig. 1a, Supplementary Data S1). Linear motifs identified by rmotif-X differed by 6–67% when the same sequences (foreground and background) were read backwards (reversed) (Fig. 1a). Interestingly, we did not observe this effect in the dataset used in the original Motif-X publication^20^. Similarly, the MoMo implementation^21^ of Motif-X did not show a positional bias and correctly identified the identical set of linear motifs when sequences were presented forward or backwards. This is possibly because of improvements to the p value calculation in the MoMo re-implementation of Motif-X. Based on these results we concluded that rmotif-X should not be used for robust analysis of linear motifs.

The field has recognized that instrumentation is becoming so sensitive that not only major products of enzymatic post-translational reactions are identified but also the products of stochastic chemical modification of amenable residues^5^. To evaluate if MoMo is robust against noise, we added a single random sequence at the beginning or at the end of the TagGraph serine-centered phospho dataset, analyzed both datasets using the default settings, and compared the results to the original. Unexpectedly, the addition of a single sequence resulted in 31% of patterns that were different between both datasets. (Fig. S1a, Supplementary Tables S1, S2). Even more surprisingly, this was only the case when the single random sequence was added at the top of the list but not when added to the end of the list of sequences (Fig. S1a). This led us to investigate the effect of sequence order. When we analyzed the same dataset with randomly reordered input sequences using MoMo default settings, only 30% of identified patterns were identical between original and reordered sequence order (Fig. 1b, Supplementary Data S2). We found this effect to be consistent across most datasets we analyzed (Fig. 1b). Based on these results we concluded that MoMo also does not support robust analyses if minor changes were made in the input data and had to be excluded.

To ensure accurate and robust motif enrichment we developed a new algorithm for RObust LInear Motif deconvolution (RoLiM) (a synopsis can be found in “Methods” and a detailed description and performance evaluation in Supplementary Text and Material). We identified the dominant cause of the positional bias in rmotif-X to be the biased selection of overlapping subsets defined by equally-enriched residues (Fig. S1b) and suspect that problems with the background frequency calculation are the origin of the sequence order bias in MoMo. Following the same proven design principle of Motif-X, we eliminated the localization bias by allowing for overlapping subsets (Figs. S1b,c), re-implemented the background frequency calculation, and increased the precision of the significance calculation. We found RoLiM to be sensitive, deterministic (Supplementary Text), and robust against sequence reversal (Fig. 1a), addition of noise (Fig. S1a), and sequence order (Fig. 1b). RoLiM has an equally low likelihood of < 0.1% to detect a pattern in a random proteome subset as Motif-X (Fig. S1d). Using this robust algorithmic framework, we then introduced additional functionality not currently implemented by similar algorithms. As many modifications, such as phosphorylation, do not only occur on a single target residue we eliminated the requirement to define a central residue. To account for the observation that the substrate specificity of protein modifying enzymes is often not defined by a specific residue but by specific physicochemical properties at a specific position, we further introduced the option to screen for enrichment of such pre-defined or custom compound residue groups sharing specific properties^30–32^ (Fig. 1c). To demonstrate the effect of compound residues on motif enrichment we applied it to a large dataset of phosphoproteome changes during epithelial-to-mesenchymal transition^33^. The two top enriched motifs without use of compound residues were highly redundant. However, accounting for similar properties combined the two top motifs into a single motif with negatively charged residues on the C-terminal side of the central serine and showed a second, highly-enriched motif with basic residues on the N-terminal side of the phosphorylated serine **(**Fig. 1d, Supplementary Table S3). Thus, in this case, accounting for compound residues allowed data-driven aggregation of similar motifs based on their physicochemical residue properties.

**Figure 2:**
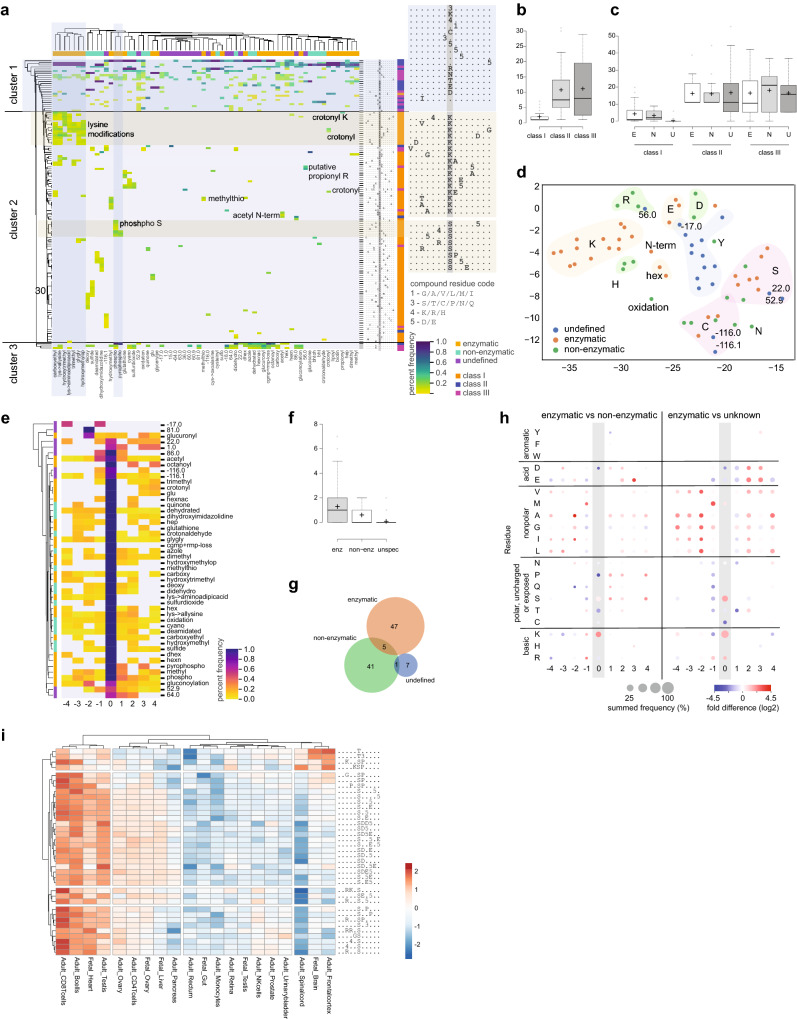
Proteome wide pan-modification. (**a**) Hierarchical clustering of all modified sequences matching all extracted motifs. Motifs are identified by RoLiM for each modification separately. The fraction of sequences in each modification matching to each motif is displayed and clustered using the Euclidean distance. Only significantly enriched modification to motif matches are considered. Column grouping indicates the modification type and row grouping indicates the three motif complexity classes. (**b**) Number of modifications matching patterns of different complexity classes. (**c**) Percentage of patterns in each complexity class matched by enzymatic (E), non-enzymatic (N) or undefined (U) modifications. (**d**) t-SNE embedding of the data represented in a. Shaded areas are added manually to highlight undefined modifications and modifications matching motifs with the indicated central residues. Overlapping areas indicate modifications matching to motifs containing either central residue. Numbers denote mass shifts of select undefined modifications. (**e**) Positional load of modification associated motifs. The frequency at which a position is identified as overrepresented in the motifs is plotted and rows clustered using the Euclidean distance. (**f**) Number of modifications for a specific modification type matching Class I patterns. (**g**) Tissue specificity of phosphorylation motifs. Ln(x + 1) transformed, scaled and centered summed counts of sequences identified in each tissue and matching each pattern are clustered and plotted.

After confirming that our algorithm is robust against residue position and sequential sequence order, particularly in large datasets with many specificities, we explored its capabilities on the largest dataset of mixed post-translational modifications assembled to date. We analyzed 49,831 sites modified by one of 32 enzymatic, 19 non-enzymatic modifications or 17 unclassified mass changes identified by TagGraph^14^ in the human draft proteome dataset^26^ consisting of 30 adult and fetal human tissues. The hierarchical and sequential clustering built into RoLiM facilitates visualization and interpretation of large complex datasets (Fig. 2a). In this first proteome-wide, pan-modification, global linear motif analysis several striking observations could be made. Even in this non-enriched global analysis, the sequence preferences of several kinase classes are clearly identified (e.g. cyclin dependent and MAP kinases [xxxxSPxxx]; casein kinase 2 [xxxxSxxEx; xxxxSxxxD/E]). As expected, the motifs identified at higher frequencies are not as rich, showing fewer significantly enriched positions than commonly found in datasets following phosphopeptide enrichment. We also find several instances of enriched residues in positions − 7 and + 7 from the modified residue. While less commonly described, these could correspond to residues two helix loops apart facing the same helix interfaces as the modified residue. Interestingly, serine phosphorylation occurs in sequence contexts that are restricted to this common and highly-regulated modification. Similarly, the sequence determinants for enzymatic lysine modifications are largely distinct.

In addition to classic motifs comprised of a central modified residue and neighboring enriched residues (defined here as Class I) RoLiM also found enrichment of poorly defined sequence contexts comprised of only the central residue (Class II) or enriched residues flanking a non-enriched modified residue (Class III). Hierarchical clustering mostly separated Class I from Class II and III patterns. Class I patterns are restricted to few modifications while Class II and III patterns are associated with many different modifications. (Fig. 2b). As expected, we found all three modification groups, enzymatic, non-enzymatic and undefined, to be equally associated with ill-defined Class II and III patterns. Interestingly however, only undefined modifications where almost never described by Class I patterns while they matched non enzymatic and enzymatic modifications with similar frequency (Fig. 2c). These observations may be confounded by differences in the prevalence at which modifications and mass shifts were observed. More frequently observed modifications will provide a larger sequence diversity enabling extraction of more defined and robust motifs.

With this caveat in mind, RoLiM deconvolution of the pan-modification proteome-wide dataset further enables identification of sequence context similarities between uncharacterized and known modifications using t-SNE dimensionality reduction. It is noteworthy that most uncharacterized modifications group together while enzymatic and non-enzymatic modifications group primarily based on the modified residue. Select mass shifts for uncharacterized modifications group together with known modifications suggesting that they occur in a more defined sequence context (Fig. 2d). This is further supported by analyzing the importance of individual positions for defining the sequence context around a modification. Interestingly, summative analysis of the RoLiM derived motifs highlights enzymatic modifications that lack a defining central residue in up to 70% of the associated motifs (Class III) (Fig. 2e). Closer inspection of these modifications, including glucuronylation, gluconoylation and acetylation, shows that these can occur independent from the side chain at the N terminus. Thus, RoLiM enables for the first time, the identification of sequence determinants underlying N-terminal modifications in an unsupervised way. It is important to note that the enriched motifs will reflect sequence requirements of the modifying enzyme as well as the sequence context of the terminus itself, such as translation initiation sites, or the sequence specificity of a protease^6,34,35^. Other enzymatic modifications, like cgmp + rmp-loss and dhex O-glycosylation are almost exclusively identified by Class II patterns showing no or little sequence preference outside the modified residue in line with the lack of established consensus sequences for these modifications. Enzymatic modifications are on average characterized by significantly more Class I patterns than non-enzymatic and undefined modifications (Fig. 2f). Intriguingly, Class I patterns almost exclusively characterize enzymatic or non-enzymatic modifications with only 5 patterns matching both (Fig. 2g). A closer evaluation reveals that nonpolar and aliphatic residues appear to be far more prevalent in the context of enzyme driven modifications than non-enzymatic or unknown modifications with the − 2 position being particularly enriched (Fig. 2h). In contrast, polar and uncharged residues appear to be favourable in the − 1 and − 2 positions neighbouring non-enzymatic and unknown modifications. Interestingly, basic residues appear to be preferred N-terminally of non-enzymatic modifications while acidic residues are predominantly enriched C-terminally of enzymatic modifications. The latter is possibly driven by the abundance of kinases recognizing motifs with acidic residues in position 1–3 (Fig. 2h).

Lastly, we investigated if RoLiM could be used to identify tissue specific motifs in this human proteome dataset. To ensure sufficient sequence diversity in this non-enriched dataset we limited the analysis of tissue specificity to phosphorylation and used the ability of RoLiM to first identify patterns enriched in each tissue subset and then match sequences from each subset against all identified patterns enabling true multi-sample comparison. Hierarchical clustering (Fig. 2i) showed that in this dataset, adult immune cells and fetal heart had the most sequences matching all patterns. It is unclear if this is a biological reflection of the signaling activity of these tissues, or an analytical artefact of this particular re-analyzed study. RoLiM analysis did also show a moderate enrichment of acidic casein kinase like motifs in fetal and adult ovary, in adult CD4 T-cells, as well as in fetal liver. A prominent, strong enrichment of threonine and proline directed serine (KxSP, KxxSP) phosphorylation was detected in fetal and adult brain (Fig. 2i).

To further demonstrate the power of RoLiM to identify biologically-relevant information, we analyzed a large-scale phosphorylation dataset reported by Olsen et al.^36^. The authors studied the effect of 30 kinase inhibitors on ten protein kinases in the epidermal growth factor (EGF) signaling pathway after EGF stimulation in RPE1 cells. RoLiM analysis of the 1275 phosphorylation sites (Supplementary Data 6 in^34^) reported to be regulated in at least one drug treatment condition revealed 27 overrepresented motifs (Supplementary Table S4). Visualization using RoLiMviz, and based on the average abundance of peptides contributing to each motif, revealed clusters defined by kinase inhibitors, suggesting that these patterns match likely substrates targeted by the individual kinases (Fig. S2a). Our results also concur with the reported kinase motif enrichment analysis on known substrates and kinases reported in the study by Olsen et al. As expected, EGF receptor inhibitors largely shut off the signaling pathway. Phosphosite motif abundance is very similar to that in cells with no EGF stimulation (no serum). In addition to this, motifs identified with RoLiM matched known substrate motifs for serine, threonine and tyrosine kinases, including EGFR, MEK, GSK3, and Akt.

**Figure 3:**
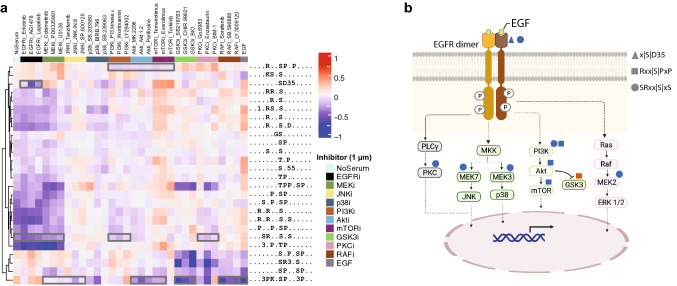
EGF pathway inhibition analysis. (**a**) Enriched phosphorylation patterns identified by RoLiM. Phosphosites analysed in RoLiM lacked motifs matched to curated kinases in Olsen et al. data (see “Methods”). The abundance of each pattern (calculated as the average abundance of all phosphosites, is shown for the different experimental conditions—RPE1 cells without EGF stimulation (No serum), with EGF stimulation (EGF), and treated with 1 µm of a panel of inhibitors targeting 10 kinases in the EGF pathway. (**b**) A scheme of the EGF pathway showing EGFR and downstream kinases targeted with specific inhibitors in Olsen et al. Broken lines denote a series of steps involving other proteins and phosphosite abundance changes are depicted in blue (downregulation) and red (upregulation).

The kinase motif enrichment approach employed in the original study could not link kinases to identified phosphosites that do not follow the curated kinase motifs, and the authors did not further investigate these. We next performed RoLiM analysis on all 1133 phosphosites not matched to a known kinase. Most of the 26 enriched motifs (Fig. S2b, Supplementary Table S4) matched kinase inhibition patterns shown in Supplementary Fig. S1a, indicating that these substrates could also be targeted by EGFR or other downstream kinases in the EGF signaling pathway cascade. We also identified 3 distinct patterns that revealed new insights not described by Olsen et al. (Fig. 3a). Inhibition of EGF signaling via the PI3K/Akt/mTOR route led to downregulation of phosphosites that conform to an Rxx|S|PxP motif (Fig. 3a,b). PI3K inhibition showed the strongest reduction in these phosphosites. Till date, no substrate motif has been curated for PI3K. Upregulated phosphosites matching Rxx|S|PxP pattern in GSK3 inhibited cells implies that this motif is related to substrates targeted by PI3K/Akt/mTOR kinases. An overrepresentation of the x|S|D35 motif was consistent after EGFR inhibition. While the overrepresented 3PKx|S|Pxx3P pattern matches substrate phosphosites that are likely lost or reduced following EGFR activation.

In conclusion we have developed a new algorithm for fast and accurate deconvolution of linear amino acid motifs in large proteomic datasets. RoLiM overcomes positional biases of existing algorithms and introduces new capabilities. The algorithm does not limit motifs to a single central residue and enables identification of physicochemical properties or compound residue groups in addition to individual amino acids. It furthermore enables quantitative and comparative analysis of multiple samples or conditions. Together these advances now enable accurate and unsupervised analysis of large protein modification datasets.

## Methods

### Datasets

Published data was retrieved from the cited sources as specified.

### RoLiM algorithm description and data flow

RoLiM was implemented in Python and functions according to the workflow depicted in Supplementary Fig. S3. The implementation and extensive quality and performance evaluation of the algorithm is detailed in the Supplementary Text and Material. Briefly, RoLiM detects overrepresented (enriched) patterns of positional residues in aligned sequence data. This is accomplished by identifying statistically enriched residues in each position of the input data set. The algorithm then sequentially selects the most enriched position/residue pair and partitions the foreground data set into two subsets defined respectively by sequences including and sequences not including that pair. In the event that multiple position/residue pairs are equally, maximally-enriched, all of those pairs are selected and this partitioning is performed for each pair simultaneously. In some cases, a single sequence may include multiple maximally-enriched pairs. Such a sequence would be included in all subsets matching those pairs and removed from the remainder subset. This set reduction operation facilitates the detection of many over-represented residues, even in cases where one or more positional residues are so abundant that they suppress the statistical significance of other positional residues when p-values are calculated simultaneously. RoLiM then recompiles the statistical background and recomputes the foreground statistics for each new subset. In this manner, the algorithm recursively constructs a set of motifs consisting of one or more fixed position/residue pairs, where each fixed pair represents a statistical over-representation in a subset of the aligned input sequences. The relationship of all sequences and motifs can be visualized by hierarchical clustering supporting the easy evaluation of motif prevalence and identification of motif families.

All RoLiM analysis utilized the default p-value cutoff of 0.001 with Bonferroni correction and a minimum support sequence threshold of 20 sequences. These settings were selected for comparability to the default settings used by Motif-X. Custom defined compound residue groups can be separately enabled (see Supplementary Text and Material). Unless specifically stated, default settings were used for all analysis.

### RoLiM visualization application (RoLiMviz)

Following analysis by RoLiM the results can be further visualized and quantitatively analyzed using the RoLiM visualization (RoLiMviz) Shiny application^37^. RoLiMviz provides logomaps of the identified motifs and aggregates the sample specific sequence intensities matched to patterns. These averaged abundances are then clustered and plotted as a heatmap to support quantitative comparison of pattern prevalence across analyzed conditions.

### Motif-X

The original Motif-X implementation and web interface is no longer available and could not be evaluated.

### Rmotif-X

All Motif-X analyses were performed using the rmotif-X re-implementation (R package rmotifx v1.0) of the original Motif-X algorithm^25^. Default settings were used in all cases, and included a p-value cutoff of 1e−06, and a minimum motif frequency threshold of 20. For most datasets, the central residue selection was ‘S’, except for the TagGraph datasets where the central residue was adjusted accordingly (see Table S1 and Supplementary Data S1). Unless specifically stated, default settings were used for all analysis.

### MoMo

All MoMo analyses were performed using the web tool available at: https://meme-suite.org/meme/tools/momo, v5.3.3^21^. Unless specifically stated, default settings were used for all analysis.

### Original vs. reversed sequences

In order to evaluate the positional (residue location) bias in rmotif-X, MoMo, and RoLiM, we compared the motifs detected in several data sets with their original (forward) sequence orientation as well as with an inverted (reverse) sequence orientation. In order to compensate for the reversal of foreground sequences, it was also necessary to invert the sequences comprising the background data set. The same foreground and matched background set was analyzed with each algorithm. Default settings were used for rmotif-X. For Momo, default settings were used with the exception of providing the forward/inverse background (extract the set of background peptides from the context sequences = TRUE), minimum motif frequency threshold was set to 20, and width of motif was adjusted to be the same as the length of sequences in the foreground (13 for Pease et al., dataset and 15 for all other datasets). For selections not stated, default settings were maintained. RoLiM default analysis settings were used, except for adjusting the width of motif to be the same as the length of sequences in the foreground (13 for Pease et al., dataset and 15 for all other datasets).

Motifs detected from reverse foreground were reversed with the function stri_reverse from the R package stringi v1.5.3 before comparing to the ones detected from the original foreground.

Venn diagrams were generated with the R package eulerr (v6.1.0).

Lollipop plots were prepared with the ggplot2 (v3.3.3) R package. The proportion of overlap shown in the lollipop graph was calculated as follows: number of the same motifs found in both forward and reverse foregrounds/sum of all unique motifs found in both foregrounds.

### Original vs. reordered sequences

To evaluate the positional (sequence location) bias in MoMo, for each dataset (TagGraph, Ochoa et al., Pease et al., and Stukalov et al., respectively)^14,27–29^, we created an additional dataset with a randomized order of the original foreground sequences in base R, and analyzed both original and randomized foregrounds in MoMo and RoLiM. We calculated the overlap of motifs detected in original and in randomized foregrounds.

Default settings were used for analyses in MoMo and RoLiM. For each algorithm, the width of motifs were adjusted according to the length of sequences in each dataset.

### Dataset composition bias

We investigated sequence location positional bias and sensitivity to dataset composition in MoMo and RoLiM. Using the TagGraph serine-centered phosphorylation dataset (dataset 1), we generated a new dataset for the TagGraph data by adding one random sequence centered at S (KDCAIQQSQYNVPYY) at the beginning of the original dataset (dataset 2) and last position (dataset 3) of the original sequence list. Each dataset was analysed in MoMo and RoLiM using the default settings and a window width of 15. The overlap of motifs detected for all three datasets in each algorithm was calculated.

### In silico demonstration of positional bias

To demonstrate the positional bias of the original Motif-X algorithm we constructed an artificial data set of 39 sequences, each 15 amino acids in length. This data set was formatted such that 19 sequences contained aspartic acid residues in the first position, serine residues in the central position, and randomly selected amino acids in all other positions. Another 19 sequences contained aspartic acid residues in the last position, serine residues in the central position, and randomly selected amino acids in all other positions. Finally, a single sequence contained aspartic acid in both the first and last position, serine in the central position, and randomly selected residues in all other positions. A second data set was derived from this data set by retaining the same structure, but inverting the order or each sequence.

These data sets were then analyzed using the rmotif-X implementation of the original Motif-X algorithm as well as RoLiM. Default settings were used for Motif-X, including a support level of 20 sequences. Default settings were also used for RoLiM, except that the minimum number of support sequences was increased to 20 sequences.

In this configuration, the data sets contained two enriched positional residues defined by an aspartic acid in either the first or last position. Furthermore, the statistical properties of these enriched positional residues are identical due to the fact that they consist of the same amino acid represented at the same frequency in a fixed-size sample. However, these enriched motifs are not mutually exclusive due to their common dependence on the single sequence containing aspartic acid in both the first and last position. Sequential detection of these enriched motifs leads to this common sequence’s capture by the first motif during the set reduction operation employed by both tools. This in turn depletes the frequency of the second enriched motif below the threshold for support level enforced by the settings supplied to both tools. RoLiM does not discriminate between statistically identical, maximally enriched positional residues, instead selecting all maximally enriched positional residues simultaneously, giving rise to a branch point in the data flow of the algorithm. On the other hand, Motif-X always selects a single maximally enriched positional residue, even in cases where multiple positional residues are statistically identical. This test served to elucidate the undocumented behavior of the Motif-X algorithm with respect to the order of selection with respect to the position of those statistically identical residues.

### False positive analysis

To assess the frequency at which RoLiM and Motif-X detect false positives, a number of artificial data sets were produced by randomly sampling 15 amino acid sequences from the Swiss-Prot human proteome. Due to the fact that Motif-X requires the user to specify the central residue of the foreground data set, this was repeated for all possible central residues in the proteome in order to avoid any context-dependent effects. To further account for the contributions of sample size and random variation to the results, sample sizes of 100 sequences and 10,000 sequences were used, and each combination of central residue and sample size was replicated 100 times by the same process of random sequence selection. This yielded a total of 4000 samples.

These samples were then analyzed by RoLiM and Motif-X using the default settings for each tool. A corresponding background data set was selected for each foreground data set consisting of all sequences from the SwissProt human proteome containing the same number of residues as well as the same central residue. Due to the random selection of foreground sequences directly from the background context, the presumption of this experiment was that the foreground data sets should closely resemble the background data sets at a statistical level. Therefore, neither tool should detect enriched motifs in any of these foreground data sets.

The frequency of false positives was estimated as the fraction of the total number of samples for which either tool detected at least one enriched motif. The results of this experiment were visualized in the form of a bar plot produced using the Matplotlib Python library.

The background data set for each sample was generated by extracting all subsequences of the same length as the foreground sequences from the SwissProt human proteome, where the central residues of the background and foreground sequences were the same.

### TagGraph pre-processing

Aligned sequences for the TagGraph analyses were prepared by mapping the protein identifiers and modification positions provided with the data set to the SwissProt human proteome and retrieving the residue at each position along with the seven residues flanking that position in each direction. The resultant set of sequences was then de-duplicated at the level of protein, position, and modification, only retaining multiple copies of an aligned sequence if each copy differed with respect to one or more of those properties.

In a small number of cases, the modified amino acid represented in the TagGraph data set was inconsistent with the amino acid at the protein position from the same row of the data set. These cases were excluded from subsequent processing and analysis.

Due to the large number of modifications supported by a very small number of sequences, we then filtered the TagGraph data set by retaining only modifications with at least 50 occurrences in the data set. The remaining aligned sequences were then partitioned based on the modification specified in the data set and saved to separate text files for further analysis using RoLiM and Motif-X.

### TagGraph pan-modification analysis

The motifs detected by RoLiM in each modification-specific subset of the TagGraph data set were concatenated into a single non-redundant set for simultaneous comparison to all modifications contained in the data set. A frequency matrix was then generated by calculating the number of sequences in each modification subset which exactly matched the fixed positions of each motif. To account for the wide range of modification frequencies in the data set, p-values were calculated for these frequencies and only significantly overrepresented frequencies were retained. Significant frequencies were then converted to percentage form based on the total sample size for each modification in order to facilitate comparison between modifications. Modifications were then assigned to categories based on their mechanism of regulation (enzymatic, non-enzymatic, or undefined).

Significant motif percentage frequencies for each modification set were compared by clustering the percentage frequency matrix with SciPy’s hierarchical clustering using the Euclidean distance metric and average cluster distance linkage. The results of this clustering were visualized using the Seaborn Python package’s clustermap function. Both modifications and motifs were clustered, and modification categories were incorporated as a color bar along the x-axis of the plot.

The significant percentage frequency matrix was also clustered and visualized using t-SNE using the TSNE function from the Scikit-learn Python package’s manifold module. Parameters used for this clustering included a perplexity of 15, a fixed initial random state, and 5000 iterations. Clusters were visualized using the scatterplot function from the Seaborn Python package.

To better understand the global features of the TagGraph data set, the positional load of motifs matching modifications with 100 sequences or more was analyzed in greater detail. Positional load was calculated as the percentage of motifs containing any fixed residue in a specific position for each modification. The matrix of these positional load calculations was then clustered using SciPy’s hierarchical clustering with a Euclidean distance metric and average cluster linkage, and visualized using Seaborn’s clustermap function. A color bar indicating the mechanism of regulation of each modification was also included in on the y-axis of this plot.

Further analysis of the global composition of the TagGraph data set was performed by comparing the frequency of specific fixed positional residues of the motifs detected by RoLiM with respect to modification type. This was accomplished by partitioning the modifications according to their regulatory type (enzymatic, non-enzymatic, or undefined) and calculating the frequency of each fixed positional residue occurring in all motifs detected in the sequence subsets matching modifications of that type in the TagGraph data set. Following this, the absolute frequencies were determined and then converted to percentage frequencies based on the total.

Differential values were then computed as the base-2 logarithm of the ratio of the fold difference for any two modification types [e.g. log2(Enzymatic/Non-Enzymatic)]. Distinct values were assigned for the three special cases where a fixed positional residue was present for one modification type but not for the other, and where a fixed positional residue was not found for either modification type.

These global residue composition values were then plotted as a custom dot matrix plot using the Matplotlib Python plotting library. Log-transformed fold differences were encoded using a diverging color map, while point size was used to encode the combined percentage frequency of the motifs from both modification types relative to the total number of sequences corresponding to each modification type from the TagGraph data set. Residues were sorted and partitioned along the y-axis of the plot on the basis of structural and electrochemical properties in order to elucidate any patterns in the results.

### TagGraph pan-tissue phosphorylation motif analysis

All sites classified as ‘phospho’ were extracted from the TagGraph dataset and the sequence window surrounding the modified residue derived from UniProt. For each tissue all identified sequences (intensity > 0) were extracted, prepended with the tissue name, and sequences from all tissues concatenated to a multi-sample pre-aligned RoLiM input file. RoLiM analysis was performed using default settings with a window width of 11 and elimination of sequence redundancy at the sequence level. From the result summary the number of sequences matching a pattern was summed for each pattern and the resulting matrix was ln(x + 1) transformed, rows centered and unit variance scaled before clustering and plotting.

### EGF pathway inhibition analysis

Using a large-scale mass spectrometry-based proteomics workflow, Olsen et al.^36^ studied the effect of a panel of 30 kinase inhibitors on ten key protein kinases in the epidermal growth factor (EGF) signaling pathway. From the 62 described experimental conditions in RPE1 cells, the authors report 1275 phosphorylation sites that were regulated in at least one condition (Supplementary Table 6 in Olsen et al.). This file contained average phosphosite intensities of six independent measurements from the 62 experimental conditions. We analyzed the results reported by Olsen et al. in RoLiM. To further explore the capability of RoLiM, the data was filtered to (i) remove patterns matched to known substrates and individual kinases, and (ii) restrict the analysis to conditions using 1.0 µM inhibitor.

RoLiM default settings were used for this analysis along with the following selections: p-value threshold of 0.001, minimum occurrence at 6, and position specific background set to ‘false’. Further analysis with RoLiMviz provided heatmaps to compare patterns enriched in different experimental conditions.

## Supplementary Information

Supplementary Information 1.

Supplementary Information 2.

## Data Availability

All data generated or analyzed during this study are included in this article and in its Supplementary Information files.

## Code availability

Source code and supporting files are provided in a GitHub repository (http://github.com/LangeLab/RoLiM). For convenient use we provide a streamlined web interface at http://www.langelab.org/rolim.
