# Supplementary material for: Robust unsupervised deconvolution of linear motifs characterizes 68 protein modifications at proteome scale
Source: Sci Rep. 2021 Nov 18;11:22490. doi: 10.1038/s41598-021-01971-3 (PMC8602328; doi:10.1038/s41598-021-01971-3)
Supplement: Supplementary file 2 — Supplementary Information 2. [file 41598_2021_1971_MOESM2_ESM.pdf]

Robust unsupervised deconvolution of linear motifs characterizes 68 protein modifications at proteome scale

Theodore G Smith, Anuli C Uzozie, Siyuan Chen, Philipp F. Lange*

Supplementary Text and Material

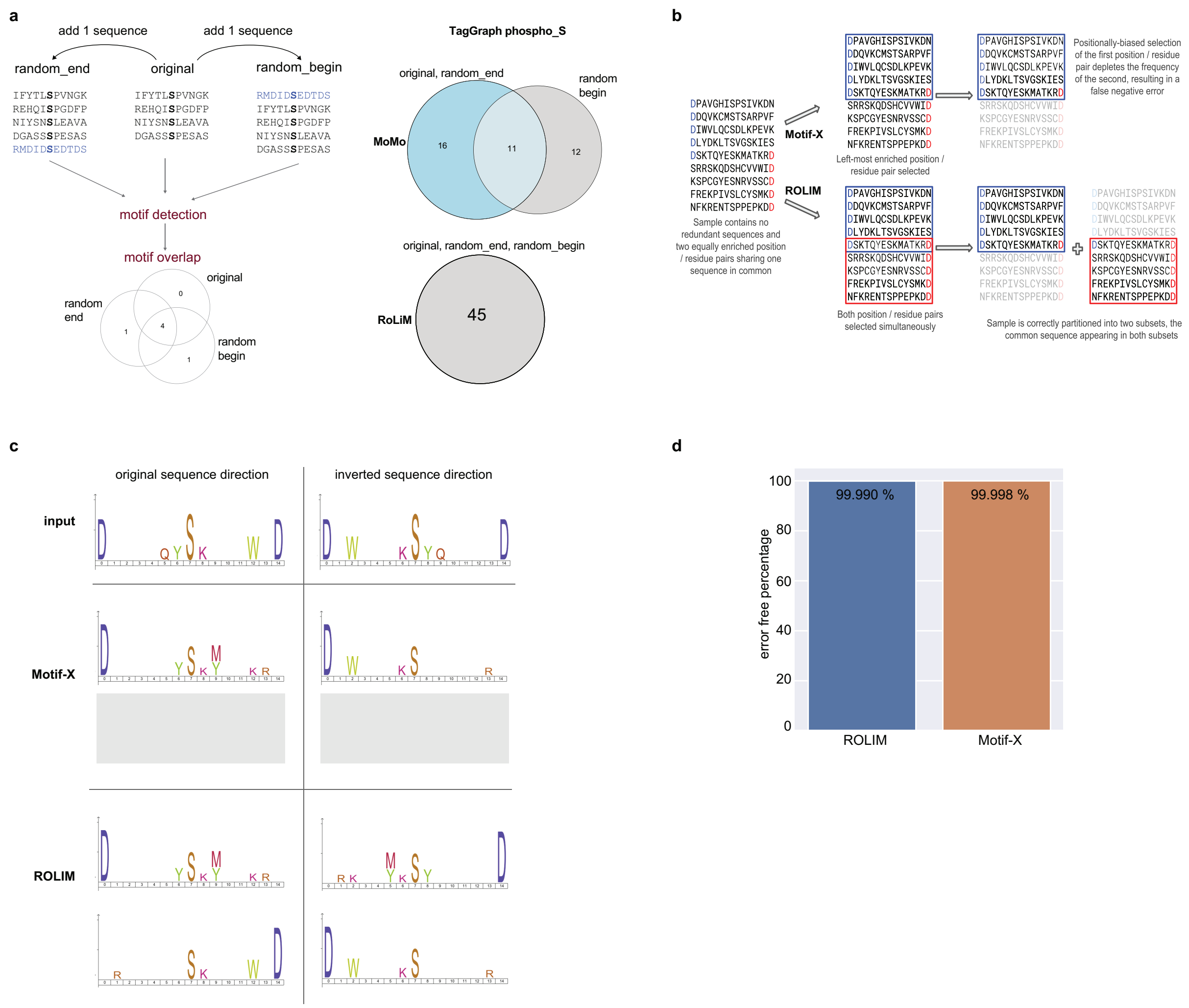

Figure S1. Positional and sequence composition bias analysis between algorithms. | **a.** Overlap between motifs identified by rmotif-X, MoMo, and RoLiM in original sequence datasets compared to same datasets with a random sequence at the end (random_end) or at the beginning (random_begin) of the sequence list respectively. **b.** Schematic depiction of the effect underlying the positional bias in Motif-X and solution implemented by RoLiM. **c.** Sequence logos of motifs identified on a synthetic dataset demonstrating the positional bias in Motif-X and accurate identification by RoLiM. **d.** Percentage of sequence sets containing no true enriched motifs which returned no false positive motifs as analyzed by RoLiM and Motif-X.

a

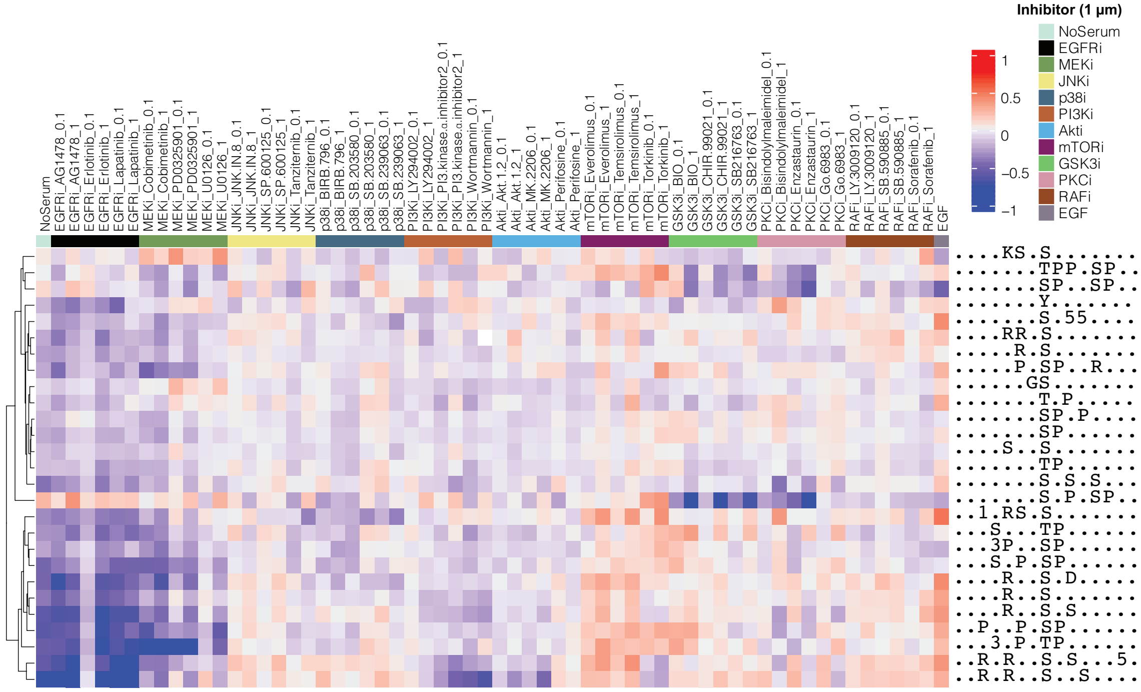

b

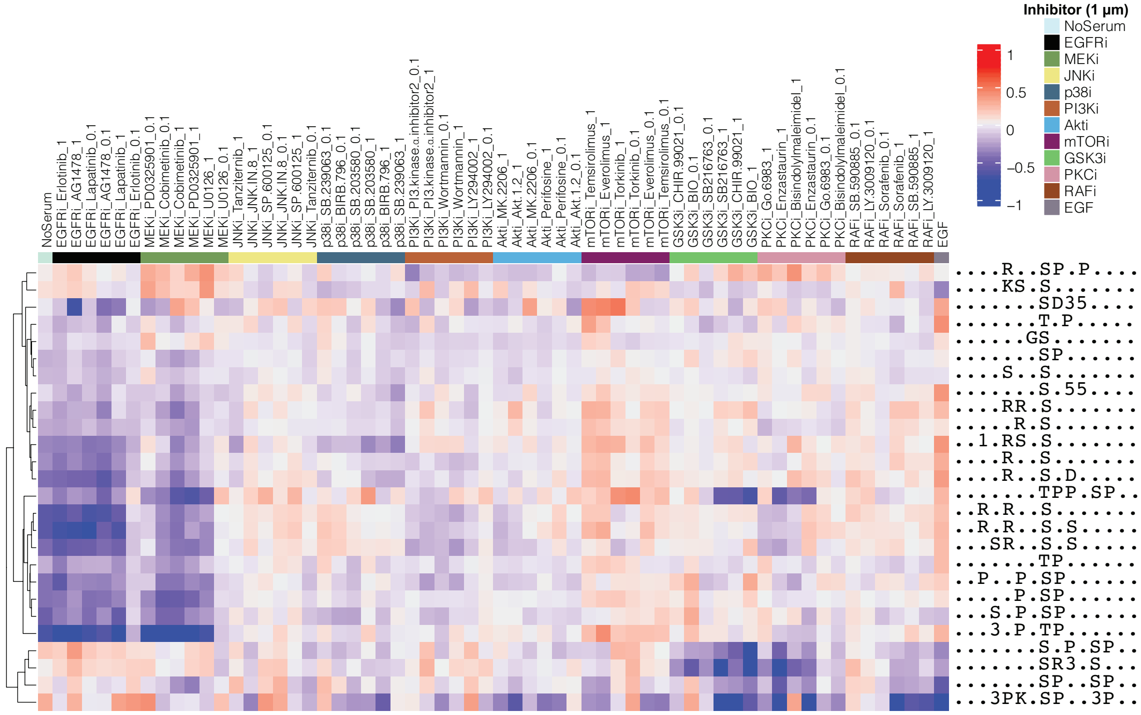

Figure S2. Enriched phosphorylation patterns identified by RoLiM after EGF pathway inhibition. | **a.** The abundance OF phosphosites matching each motif is shown for the different experimental conditions - RPE1 cells without EGF stimulation (No serum), with EGF stimulation (EGF), and treated with a panel of inhibitors targeting 10 kinases in the EGF pathway (EGFR, MEK, JNK, p38, PI3K, Akt, mTOR, GSK3, PKC, and RAF). Cells were treated with EGF prior to perturbation with the selected inhibitor at 0.1 and 1 μm respectively. **b.** Phosphosite abundance and overrepresented patterns in cells after kinase inhibitor treatment. Phosphosites analysed specifically lacked motifs matching to curated kinases in Olsen et al.,.

RoLiM algorithm

Algorithm summary

RoLiM identifies a position/residue pair that is significantly enriched relative to what is expected based on amino acid background frequencies from a context proteome. A description of the processing workflow is shown in Figure S3. The enriched positional residues detected in each branch of the process are used to define motifs. For each motif, the fold enrichment of matching sequences are provided and displayed in a sequence weblogo[1]. To provide a summarizing motif clustermap all sequences are scored against all motifs based on the BLOSUM-62 matrix and visualized as a heatmap with hierarchical clustering of rows (motifs) and columns (sequences).

Expected residue frequencies

Patterns are detected by this algorithm on the basis of overrepresented positional residues. In order to determine whether a positional residue is overrepresented, the expected frequency of that residue in a data set of a given size must first be estimated. This is accomplished through the compilation of a statistical background to which the residue composition of the foreground data set is compared.

By default, this tool utilizes the Swiss-Prot human proteome for calculation of expected amino acid background frequencies[2]. However, user-specified background data sets are also supported. Two basic modes for expected residue frequency calculation are supported at this time.

Using the first mode, background data sets may be pre-computed by the user and supplied as either a CSV- or JSON-formatted list of expected amino acid frequencies. User-computed backgrounds may be position-specific or averaged across positions, and are iteratively recomputed as indicated in Equation S1.

To estimate expected amino acid background frequencies, the percentage frequency of each amino acid in the context proteome is calculated. These percentage frequencies are then stored in a vector of length r, where r is the number of distinct amino acids in the context proteome.

An essential operation of this algorithm is the identification of a position/residue pair that is significantly overrepresented relative to what is expected based on amino acid background frequencies from a context proteome. Sequences featuring this overrepresented position/residue pair are isolated and removed from the remainder of the foreground dataset, thus eliminating said residue from the foreground. It is therefore necessary to compensate for this removal by eliminating that residue from the background as well.

Therefore, following each removal of a positional residue, the effective background frequencies are recalculated for each position. The calculation of the effective positional background frequency for each residue is:

Equation S1. Effective expected residue frequency calculation:

$$f_{effective} = \frac{f_{background}}{100 - \sum_{i=1}^{r} f_{background_i}}$$

f: percentage frequency of residue in background frequency data set
r: number of residues eliminated from positional foreground and background

The results of the positional background frequency adjustment are stored in a matrix of shape m x r (where m is the length of the input sequences and r is the number of distinct residues in the background data set). These position-specific, effective expected residue frequencies are used for all subsequent calculations.

This mode of operation is less computationally intensive and produces results in a shorter run time, however it is only recommended when one of two conditions are true. Firstly, this mode is useful when every position in a particular fixed-width, aligned subsequence representation of a reference proteome (i.e. every 15 amino acid sequence occurring across all proteins) can be assumed to be independent.

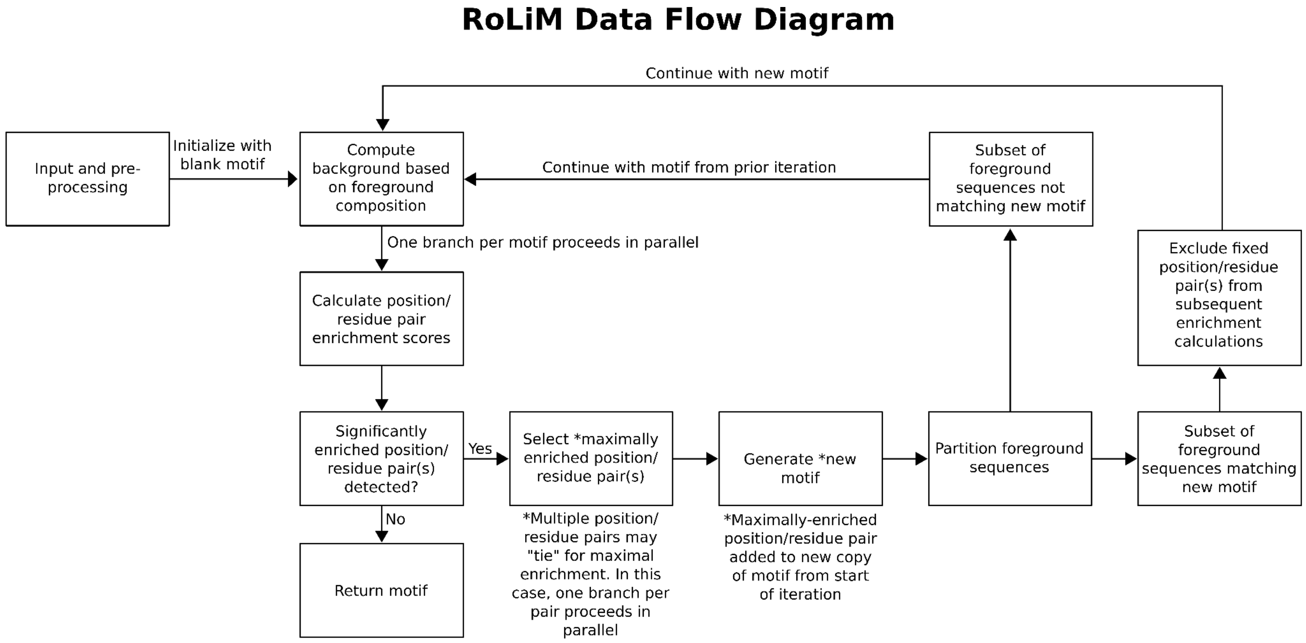

Figure S3 RoLiM Data Flow Diagram. RoLiM constructs a set of linear motifs using a recursive reduction data flow. Each iteration begins by computing a statistical background from a context data set matching the position-specific residue composition of the foreground data set. This background is used to calculate a p-value-threshold enrichment score for each position/residue pair in the foreground. If the p-value of the over-representation of at least one position/residue pair meets the significance threshold, the maximally-enriched pair is selected and sequences containing the pair are isolated from the remainder of the foreground data set.. Otherwise, RoLiM returns the set of motifs constructed during prior iterations of the branch. If more than one position/residue pairs “tie” for maximal enrichment, all maximally-enriched pairs are selected and isolated simultaneously, resulting in multiple branches of the data flow which proceed in parallel. One new motif is generated per isolated foreground subset by appending the corresponding position/residue pair to the motif from the prior iteration. A new iteration is then initiated for each motif, including both newly-defined motifs as well as the motif from the prior iteration, with the matching subset of the foreground from the prior iteration serving as the initial foreground for the new iteration.

This ensures that the set reduction operation of the algorithm does not produce specialized contexts arising as a result of correlations in the background data set rather than the foreground data set. Secondly, this mode is useful when one is interested in detecting those specialized contexts within the reference proteome, either explicitly or as a component of the patterns detected in a foreground data set. In this case, care should be taken to ensure that features associated with specialized contexts in the reference proteome are not confused with presumed biological activity in the foreground.

When neither of these conditions is satisfied, an alternative expected residue frequency calculation is recommended. This mode consists of extracting a set of fixed-width, aligned sequences from a reference proteome. By default, the Swiss-Prot human proteome is used, however the user may opt to upload their own reference proteome in FASTA format. The reference proteome should be selected with the context of the foreground in mind. Typically, the background should reflect the biological context of the foreground data set as accurately as possible, however it may be of interest in certain cases to select a background that highlights particular features in the foreground data set on the basis of statistical differences between the two.

Normally, all sequences of the specified length occurring in the reference proteome are used in order to comprehensively reproduce the position-specific context of the reference proteome. Users do, however, have the option to specify the characteristics of the sequences used by supplying a regular expression template.

These sequences are then converted to a binary tensor representation which allows for efficient calculation of the position-specific frequency of each residue. Moreover, this facilitates rapid sub-setting of the background data set in order to calculate expected residue frequencies based on the specific context of the fixed position / residue pairs associated with a particular iteration of the algorithm in the foreground.

Compound residues

A unique feature of this tool is the incorporation of compound residues. Compound residues consist of groups of two or more amino acids with similar biochemical features. These features may be structural, electronic, or functional in nature.

Inclusion of these groups allows for the detection of enriched biochemical features in a foreground data set, even when the amino acids comprising a compound residue group are not individually overrepresented.

The expected frequency of a compound residue is calculated as the sum of the expected frequencies of its constituent amino acids, as exhibited in Equation S2.

Equation S2. Effective expected compound residue frequency calculation:

$$f_{compound} = \sum_{i=1}^{c} f_{effective_i}$$

c: Number of compound residue constituents

As with individual amino acids, it is necessary to compensate these compound residue frequencies in the background for the foreground set reduction operation of the algorithm on a positional basis when the averaged background mode is utilized. To address this, the sum of expected frequencies is taken after the positional background frequency correction previously described.

Furthermore, it is possible that following the set reduction operation a subset characterized by a compound residue in a particular position may contain an enrichment of a more specific compound residue or individual amino acid(s) in the same position. To allow for further decomposition of these subsets, the effective expected residues of fixed positions are also recalculated with each iteration of the algorithm.

The calculation of the expected frequency of each residue in a fixed position consists of that residue's background frequency divided by the cumulative background frequencies of all residues possible in that position. For a single amino acid, therefore, its effective expected frequency is calculated as 100% in that position. Therefore, no further decomposition of that position will occur. This formula is expressed in Equation S3.

Equation S3. Effective expected fixed position residue frequency calculation:

$$f_{fixed} = 1.0 - \sum_{i=1}^{c} f_{effective_i}$$

Alternatively, for a compound residue, the effective expected residue frequency of each constituent amino acid (as well as of other, more specific, compound residues) will be less than 100% and proportional to that amino acid's background frequency. Therefore, if a subset characterized by a compound residue contains a disproportionately high frequency of a particular amino acid in that same position, the subset featuring that overrepresented amino acid will be isolated.

By default, the algorithm uses a list of five compound residue groups. These groups represent the historical biochemical categories of the 20 common amino acids appearing in most proteomes[1]. Proline, a nonpolar amino acid, is traditionally grouped together with the nonpolar, aliphatic amino acids. In contrast to these amino acids that are primarily buried in the core of proteins proline is commonly found to be exposed and shows equal or higher hydropathy than polar amino acids[3]. Since the level of surface exposure influences enzyme accessibility, we here classify Proline alongside polar, uncharged residues and describe this slightly altered group as "Polar, uncharged or exposed". Information regarding these compound residues including the arbitrary codes used to represent the groups by this tool, their biochemical descriptions, and the constituent amino acids belonging to each group can be seen in Table S1.

Code	Biochemical description	Amino acids
1	Nonpolar, aliphatic R groups	G, A, V, L, M, I
2	Aromatic R groups	F, Y, W
3	Polar, uncharged or exposed R groups	S, T, C, P, N, Q
4	Positively charged R groups	K, R, H
5	Negatively charged R groups	D, E

Table S5. Default compound residues. Table of default compound residue groups used by our algorithm, complete with the symbol used to encode the groups in patter string representations and visualizations, biochemical descriptions of the groups, and individual amino acid constituents of the groups.

RoLiM also allows users to submit alternative compound residue groups in the form of an optional tab-delimited file containing one compound residue group per line. The first entry in each line consists of a brief description of the group's defining characteristics. The second entry on each line consists of a list of the single-letter codes for each simple residue included in the group, separated by semi-colons (e.g. "Acidic residues D; E").

Input and preprocessing

This tool accepts a number of foreground data set formats as input. These formats include pre-aligned sequences of equal width which can be supplied either as a text file containing one sequence per line, or a FASTA file consisting of alternating identifier and sequence lines. For pre-aligned data sets, FASTA identifiers are not utilized by the tool. Alternatively, unaligned peptides may be supplied in the form of a tab- or comma-delimited text file consisting of one peptide and one protein identifier per line (in that order), or a FASTA file consisting of alternating protein identifiers and peptides.

Pre-aligned sequences may be of even or odd length, however all sequences in the input data set must be of equal length. Support for both even- and odd-length input sequences is intended to allow for the analysis of multiple types of post translational modifications as these modifications may be centered around a particular residue (such as phosphorylation) producing odd-length sequences, or around a particular bond (such as proteolysis) producing even-length sequences.

If unaligned peptides are supplied, the tool will attempt to extend each peptide using the sequence of the corresponding protein and will truncate the resulting sequences to a specified width centered on the modified residue. Peptides can be extended in the N-terminal direction, the C-terminal direction, or in both directions.

We recognize that users may attribute differing relevance to redundant sequences arising because of broader biological context beyond the range of the short, aligned sequences analyzed by this tool. Users may therefore optionally retain or eliminate redundant sequences supplied in a pre-aligned data set or produced in the alignment process. By default, any duplicate sequences included in a pre-aligned input data set are retained, and the assumption is made that the user has included those redundancies deliberately. Sequences generated by the extension and alignment utilities included with this tool are de-duplicated at the level of distinct proteomic sites by default, thus retaining only distinct molecular entities with respect to the context proteome.

Following the extension and alignment stages of preprocessing, the foreground sequence data set is converted to a binary tensor of shape n x m x r (where n is the number of input sequences, m is the length of the sequences, and r is the number of distinct residue codes). Importantly, the order of values on the r-axis of the tensor is matched to the order of values in the background frequency vector. This tensor is used for all subsequent operations on the foreground data set.

Enrichment calculation

P-values

The core operation of this tool is the detection of statistically overrepresented positional residues in a set of input sequences relative to the expected frequencies of those residues. In order to estimate significance, a p-value is calculated for each positional residue. Each individual observation of a positional residue in the aligned data set is treated as a discrete, independent event, wherein the observation of a positional residue in one input sequence does not alter the expected probability of observing the same positional residue in another sequence. P-values are therefore calculated according to the binomial distribution and reflect the probability of observing as many or more occurrences of a positional residue in a sample of size n.

To most accurately approximate these values, this tool utilizes SciPy's implementation of the regularized incomplete beta function (Equation S4) which is defined in terms of the incomplete beta function (Equation S5) and the beta function (Equation S6). The regularized incomplete beta function is the cumulative distribution function (CDF) of the beta distribution. The CDF of the beta function is related to the cumulative distribution function of the binomial distribution by the following formula where p is equal to the probability of success (the expected residue frequency in this application) and n is equal to the sample size of the subset of sequences corresponding to a particular iteration of the recursive pattern detection algorithm. Therefore, the regularized incomplete beta function directly provides the probability of observing an equal or greater number of occurrences of a positional residue based on the binomial distribution.

This approach avoids floating point rounding errors which arise when attempting to calculate the complement of the binomial CDF for very small p-values, allowing accurate calculation of p-values down to approximately 5e-324.

Due to the fact that multiple positional residue p-values are calculated and compared during each iteration, it is necessary to perform multiple testing correction when enforcing the p-value threshold. This is accomplished using Bonferroni correction, as expressed in Equation S8, where m is adjusted dynamically based on the number of residues that could be observed across all positions during each iteration[4]. Successive iterations of RoLiM represent specialized statistical contexts derived from previous iterations and, ultimately, from the initial foreground and background data sets. Therefore, the number of independent hypothesis tests performed by RoLiM is not additive across iterations but is rather maximal in the first iteration during which all position/residue pairs observed in the full foreground data set are tested. By extension, family-wise error rate is governed by the number of position/residue pairs actually observed in the foreground subset corresponding to a given iteration and is corrected for within, rather than across, iterations.

Equation S4. Regularized Incomplete Beta Function:

$$I_x(a,b) = \frac{B(x;\ a,b)}{B(a,b)}$$

Equation S5. Incomplete Beta Function:

$$B(x;\ a,b) = \int_0^x t^{a-1}(1-t)^{b-1}dt$$

Equation S6. Beta Function:

$$B(a,b) = \int_0^1 t^{a-1}(1-t)^{b-1}dt$$

- x: Upper bound of integration. Set equal to expected frequency when used to calculate binomial probability.
- a: Number of successes plus one when used to calculate binomial probability.
- b: Sample size minus number of successes when used to calculate binomial probability.

Equation 7. Binomial probability of X or More Successes:

$$Pr(X \geq k) = I_p(k+1, n-k)$$

- n: Sample size
- k: Positional residue frequency

Equation S8. Bonferroni correction:

$$\alpha_{corrected} = \frac{\alpha_{uncorrected}}{m}$$

- m: number of observed position/residue pairs

Following multiple testing correction, a maximum p-value threshold is enforced. Positional residues with p-values equal to or below this threshold are deemed significantly overrepresented, while positional residues with p-values above this threshold are deemed insignificant. The p-value threshold is set to 0.001 uncorrected by default, however this setting is parameterized and may be specified by the user. In most samples, this default settings results in a corrected p-value threshold on the order of 1e-6, which is consistent with the p-values selected for use in alternative motif detection algorithms.

Minimum frequency requirements

In addition to a p-value threshold, this tool also supports the optional enforcement of a minimum frequency threshold. Minimum frequency is enforced simply by counting the number of occurrences of a position/residue pair in the foreground data set. This threshold is parameterized and may be specified by the user according to their particular needs. By default, RoLiM enforces a minimum frequency threshold of 20 sequences. Reducing the threshold to one sequence disables the minimum frequency requirement.

Positional weighting

Enrichment scores calculated by this algorithm depend not only on the significance of the observed frequency of a positional residue in the context of its expected frequency, but also on a positional weighting factor. This term of the enrichment calculation favors low diversity positions by generating an increasing large term as the number of under-represented residues from the background data set, as well as the fold difference of their under-representation relative to their expected frequencies increases. The formula for this calculation is expressed in Equation S9.

Equation S9. Positional weighting calculation:

$$W_{position} = -log\left(1.0 - \left(\frac{\sum_{i=1}^{A}\left(1.0 - \frac{f_{observed_i}}{f_{expected_i}}\right)}{B}\right)\right)$$

- A: Number of under-represented residues in a position
- B: Number of possible residues in a position

Positional weighting serves two main purposes. Firstly, it serves to contribute additional order to the clustering of enriched positional residues in the foreground, based not only on the statistical enrichment of those residues individually but also on the structure of the data set across residues. Secondly, it serves as a potential tie breaker when two positional residues exhibit the same p-value. This option may be disabled at the discretion of the user.

Enrichment scores

Enrichment scores as calculated by this algorithm consist of the product of two terms. The first term of the enrichment score formula is the negative natural logarithm of a positional residue's p-value. The second term of the enrichment score formula is the positional weight corresponding to the residue's position in the aligned data set. If positional weighting is disabled by the user, the corresponding term in the enrichment calculation is set equal to 1. This formula is expressed in Equation S10.

Equation S10. Enrichment calculation:

$$E_{residue} = W_{position} \times -log(p_{residue})$$

Output

The default output of this algorithm consists of four main components. These include (1) a clustermap of the aligned input sequences against any patterns detected by the algorithm, (2) a set of logo maps depicting the positional amino acid composition of the aligned sequence subsets exactly matching each detected pattern, (3) a text file containing the subset of aligned sequences exactly matching each detected pattern (one file per detected pattern), (4) a pattern summary table containing each detected pattern along with a set of descriptive statistics, and (5) a tabular output file containing the sequences provided in the input data set in their original order along with additional data produced by the algorithm. Each of these output components is subsequently described in detail.

Sequence clustermap

Clustering of the input data set based on the patterns detected by the algorithm is visualized in the form of a hierarchically clustered heatmap. This heatmap consists of columns corresponding to each sequence in the aligned input data set and rows corresponding to each pattern detected by the algorithm.

Cell values represent similarity scores between each sequence and each pattern. These similarity scores represent the sum of the positional substitution probabilities for pattern and sequence residues, where positional substitution probabilities are drawn from the BLOSUM-62 substitution probability matrix. BLOSUM-62 is a broadly used substitution matrix which is computed from the pairwise substitution frequencies of each amino acid in the context of locally aligned, highly evolutionarily conserved protein sequences[3]. The similarity scores generated in this step are therefore not contingent on prior knowledge of enzyme specificity, and the resulting clustermap is minimally biased with respect to the context of proteolysis. This scoring function is represented mathematically in Equation S11 below.

Equation S11. Sequence similarity score calculation:

$$S_{sequence} = \frac{\sum_{i=1}^{m} \quad f_{BLOSUM_{s_m p_m}}}{m}$$

- m: Number of fixed positions in pattern
- $f_{BLOSUM62}$: Substitution frequency of the foreground sequence positional residue and enriched pattern positional residue
- s: Foreground sequence
- p: Enriched pattern

Clustering is performed using SciPy's hierarchical (agglomerative) clustering algorithm. Pairwise distances are computed using SciPy's calculation of the cosine similarity distance metric (Equation S12) and agglomeration order is determined using SciPy's "complete" method which employs the Farthest Point / Voor Hees Algorithm[5]. This algorithm determines clustering order by selecting the two clusters with the smallest maximum pairwise distance between the vectors in each cluster. The clustermap is then rendered using Python's Matplotlib package.

Equation S12. Cosine similarity:

$$cos(\theta) = \frac{A \cdot B}{|A||B|}$$

Pattern-matching sequence subset logo maps

Logo maps are used to visualize the amino acid distribution of each subset of aligned input sequences exactly matching one of the detected patterns. These figures are generated using the Python implementation of WebLogo[1].

Matching sequence subsets for each pattern

The complete subset of aligned input sequences exactly matching each detected pattern is provided as a text file. This decomposition of the input data set based on patterns facilitates follow up analyses on subsets of the data corresponding to features of interest based on the demands of a particular user and their analytical objectives.

Pattern summary table

A pattern summary table is included along with the outputs corresponding to each individual pattern detected by the tool. This table features a single row for each detected pattern. Each row contains a string representation of the pattern, the absolute frequency of sequences belonging to the subset of sequences defined by the pattern (Sample Frequency), the absolute frequency of sequences containing the fixed positional residue defining the pattern (Foreground Frequency), the absolute frequency of sequences in the foreground subset in which the pattern was detected (Foreground Size), and a pattern enrichment score calculated as (Sample Frequency) / (Background Frequency), where (Background Frequency) is equal to the total number of occurrences of the pattern in the context data set.

Tabular output

A summary, tabular output file is generated for each input data set supplied to this tool. This file consists of a column containing the original input sequences in their native order, a column containing the protein accession number associated with the sequence (empty if a pre-aligned data set was supplied by the user), an additional column for each position of the aligned sequence set analyzed by the algorithm, and an additional column for each pattern detected by the algorithm (populated with either a 1 if the sequence on that row exactly matches the pattern on that column, or a 0 if the sequence on that row does not match the pattern on that column).

The inclusion of this file in the output data set is intended to support the integration of this tool into a more comprehensive workflow. In essence, this file serves as an augmented version of the input data set, representing the data in its original arrangement along with the additional information generated by the algorithm.

Motif significance

The p-value threshold selected for a RoLiM analysis is applied at the level of position/residue pair frequencies observed in each iteration of the algorithm. Importantly, these p-values are not the same as those corresponding to motif enrichment in the foreground data set, nor is the p-value of a reported motif's foreground enrichment simply the product of the p-values of its fixed position/residue pairs as calculated from the iteration-specific, foreground/background context in which they were added to the motif. Position/residue pair significance is related to motif enrichment significance in the sense that applying a significance threshold at the level of position/residue pairs with respect to specialized statistical contexts in subsets of the foreground and background limits both the number and diversity of reported motifs. Enforcing a significance threshold at the level of position/residue pair enrichment does not, however, exclude motifs whose overrepresentation in the full foreground data set would be considered insignificant based on some motif-level p-value threshold. Furthermore, it does not limit the rate at which motifs evaluated as significant based on that same threshold are overrepresented due to chance. RoLiM reports the full, unfiltered list of motifs constructed by its selection of significantly overrepresented position/residue pairs within iteration-specific subsets of the foreground and background.

Algorithm performance evaluation

The accuracy of this tool was assessed through a series of analyses, each intended to evaluate a particular characteristic of the algorithm's performance. The specific objective, methods, and results of each of these experiments are discussed in detail.

As these tests were intended to evaluate the worst-case performance of the algorithm, the averaged background mode was utilized, which is susceptible to a higher rate of error as estimated in our analyses. As discussed in the description of this mode, the averaged background mode may detect enriched position-residue pairs based on true positional correlations in the background corresponding to a data set, rather than strictly on differences between a foreground data set and its background. This does not arise when the complete background data set is used due to the fact that it incorporates these correlations into its expected frequency calculations.

Methods

Algorithm settings
RoLiM settings were selected to probe the lower bounds of detection and were common across all experiments described in this Section unless otherwise noted. These settings included a p-value threshold of 0.001 uncorrected with Bonferroni correction enabled, a minimum frequency threshold of 2 sequences (functionally disabled), and position specific background calculation disabled (averaged background mode).

In silico sequence sets
Where *in silico* sequence sets were used, these sets were constructed using custom Python scripts which have been made available in the testing section of the GitHub repository associated with this tool. The non-fixed positional amino acids comprising these sequence sets were drawn randomly with the probability of drawing a particular amino acid corresponding to the expected background frequency of that amino acid in the Swiss-Prot human proteome.

Calculation of F1 scores
F1 scores were used as a metric of algorithm accuracy in several of the experiments described below. F1 scores estimate accuracy as the harmonic mean of precision and recall, and were averaged across 100 replicates for each experimental condition in all analyses. These scores were computed using Python using the formula described in Equation S13-15 and the associated code has been made available in the testing section of the GitHub repository for this tool.

Equation S13. F1 Score Calculation: $F_1 = 2 \times (\frac{Precision \times Recall}{Precision + Recall})$

Equation S14. Precision calculation: $Precision = \frac{tp}{tp + fp}$

Equation S15. Recall calculation: $Recall = \frac{tp}{tp + fn}$

- tp: Number of true positives
- fp: Number of false positives
- fn: Number of false negatives

Investigation of minimum absolute frequency
Objective
Due to the fact that this tool uses a p-value threshold to determine whether or not a particular positional residue is significantly enriched, the true positive rate in samples containing only sequences featuring a single pattern is anticipated to be predictable and ordered based on the expected background frequencies of each residue. Relative to all residues in the background frequency set, the least common residue will be counted significant in the smallest sample where one hundred percent of the sequences in that sample contain said residue in the same position. Conversely, the most common residue will require the largest sample containing only sequences featuring that residue in the same position in order to be counted as significantly enriched. All other residues fall between the least common residue and most common residue in terms of the minimum absolute frequency at which they will be detected as significantly enriched.

However, in samples containing sequences featuring more than one position, residues in other positions may contribute to the overall error rate in two ways. Firstly, other positions may contribute false positives to the outcomes of an analysis. This occurs when a position contains a spurious over-representation of a particular residue arising either as a result of random selection during construction of an artificial data set, by coincidental co-occurrence of a particular residue in a specific position due to chance rather than biological activity, or as a result of an off-target biological activity which generated a true enrichment of a positional residue but which is not of interest with respect to a particular analysis.

Secondly, these same sources of error may contribute false negatives to the outcomes of an analysis if the spuriously enriched positional residues occur in sequences also featuring the pattern of interest. Due to the set reduction operation of this algorithm, if an erroneous pattern is detected prior to the pattern of interest under these circumstances, the number of sequences corresponding to the pattern of interest is depleted thus diminishing the likelihood of its detection. If represented at equal or greater frequency than the erroneous pattern, this error will not be encountered if the pattern of interest is the least abundant residue in the background data set. This is due to the fact that its lower expected frequency will produce a smaller p-value resulting in that positional residue being selected first. Therefore, for a sample in which every sequence contains the lowest expected frequency residue in a particular position, this source of error should be impossible as the frequency of any other

enriched residue cannot possibly exceed the frequency of the lowest expected frequency residue. On the other hand, this scenario is most likely to be observed when the pattern of interest corresponds to the highest expected frequency residue.

Based on these factors, the highest overall error rate in a simple sample containing a single enriched positional residue is expected to occur when that residue has the highest expected frequency, the lowest overall error rate is expected to occur when that residue has the lowest expected frequency, while samples containing any other enriched residue are expected to exhibit overall error rates between the two in proportion to the expected frequency of the enriched positional residue. Therefore, to assess the lower bound of detection of this algorithm in terms of absolute pattern frequency, samples containing the lowest and highest background frequency residues were analyzed.

Methods

Randomly selected samples containing between 1 and 20 sequences were incrementally generated, with each sequential iteration increasing the number of sequences by 1. From these random samples, two sample variants were generated by substituting all residues in a single position with either the least abundant amino acid, tryptophan, or the most abundant amino acid, leucine, based on Swiss-Prot background frequencies. Substituting the amino acids into otherwise identical sets of sequences ensured perfect comparability of error rate between the two data sets. 100 replicates were generated and analyzed for each combination of sample size and fixed residue and the number of true positives, false positives, and true negatives were recorded using the output of each analysis. Precision and recall were computed from these outcomes, which were in turn used to calculate an F1 score for the results associated with each replicate. The mean F1 score for each combination of sample size and amino acid was calculated across replicates and plotted on a single axis.

Results

Extremely similar results were observed for both the most and least abundant amino acids with respect to the lower bound of detection in terms of absolute pattern frequency, as shown in Figure S4. In both cases, the pattern of interest was detected with 50% accuracy at a sample size of 4 sequences, and accuracy stably exceeding 95% when the sample size was greater than or equal to 7 sequences.

These results indicate that the default settings of the algorithm are sufficiently sensitive to detect over-representation of even the most common residue, while still specific enough to reject the types of errors that are likely to be observed under the same conditions.

Investigation of minimum percentage frequency

Objective

While the previous analysis served to evaluate the performance of the algorithm on the simplest possible pattern-containing data sets, it is rare that every sequence in a biological sample corresponds to an individual activity represented by a single enriched positional residue. With that in mind, this Section addresses the detection of a single, enriched pattern against a background of noise.

In addition to the errors discussed in evaluating the minimum absolute pattern frequency detectable by this tool, this sample configuration is susceptible to three other sources of error. Firstly, false negatives may arise if the percentage frequency of sequences exhibiting the pattern of interest is not sufficiently high for the positional residue to pass the p-value threshold enforcement of the algorithm.

Secondly, the set reduction operation of the algorithm may give rise to a false negative by depleting the frequency of sequences exemplifying the pattern of interest in the event that a false positive is encountered at an earlier stage of the recursive dataflow. While a sub-type of this error was already discussed at length, the description must be extended to patterns defined by a position containing tryptophan (the least abundant residue based on Swiss-Prot background frequencies) when the percentage frequency of the pattern is less than 100%. This is due to the fact that it is possible for a pattern defined by a higher-expected-frequency positional

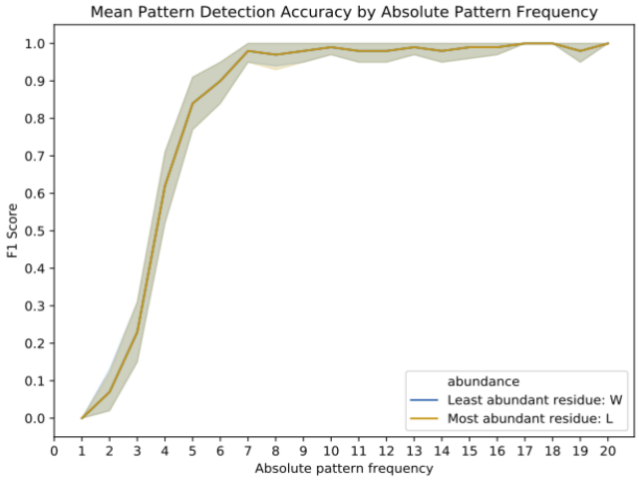

Figure S4. Mean Pattern Detection Accuracy by Absolute Pattern Frequency. Mean F1 scores for samples of size increasing from 1 sequence to 20 sequences, within which every sequence contained a particular fixed positional residue. These residues were selected to represent the lowest and highest expected frequency amino acids based on background frequencies calculated from the Swiss-Prot human proteome. These are predicted to be detectable and the lowest and highest absolute frequencies, respectively. The blue line represents results for the lowest expected frequency residue (tryptophan), and the orange line represents results for the highest expected frequency residue (leucine).

residue to produce a p-value lower than the tryptophan-defined pattern in the event that its percentage frequency sufficiently exceeds that of the tryptophan-defined pattern.

Thirdly, one of the novel features of this pattern detection algorithm is the implementation of compound residues, which aggregate multiple individual amino acids contained in the same position into larger groups based on common biochemical features shared by the amino acids. With this feature enabled, analysis of a sample containing a single pattern at lower than 100% frequency may produce a single false negative accompanied by one or more false positives. This form of error arises when the pattern of interest is aggregated into a compound residue group due to the coincidental over-representation of one or more other constituent(s) of the same compound residue in that position. While the algorithm does allow decomposition of compound residues into simpler compound residues or individual amino acids, it is possible for this process to fail to resolve the pattern of interest if the coincidental enrichment of the other constituent(s) happens to be proportional to the residue of interest.

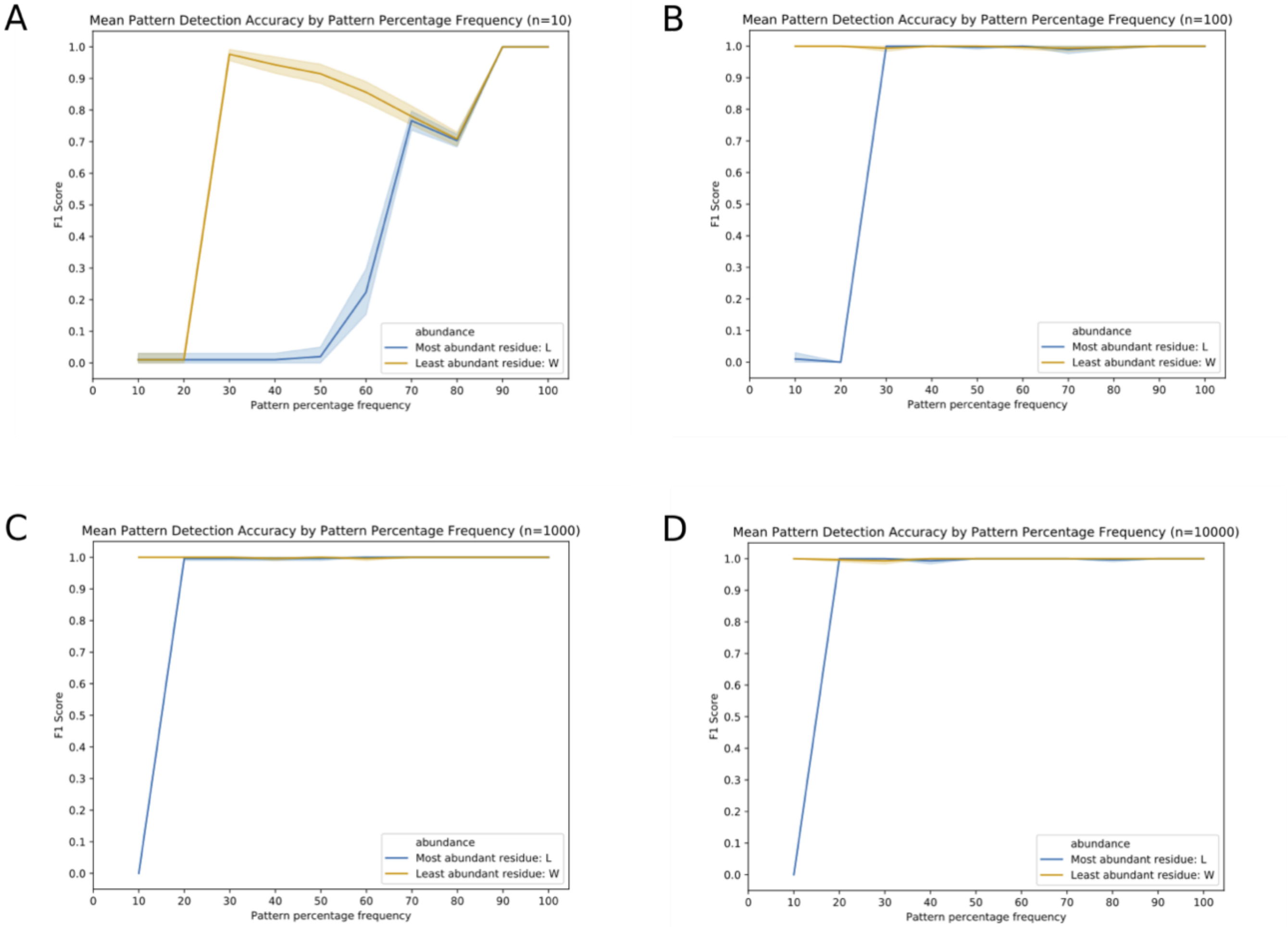

Figure S5. Mean Pattern Detection Accuracy by Pattern Percentage Frequency. F1 scores averaged across replicates showing the accuracy of the algorithm on samples of various sizes, incrementing the percentage frequency of the pattern of interest from 10% to 100% of the total sample size in steps of 10%. Total sample size was held constant for in the datasets corresponding to each subfigure. Therefore, as pattern percentage frequency was increased, noise sequence frequency was decreased in equal proportion. (A) Results for samples containing a total of 10 sequences. (B) Results for samples containing a total of 100 sequences. (C) Results for samples containing a total of 1,000 sequences. (D) Results for samples containing a total of 10,000 sequences.

Methods

For these experiments, four data sets with a fixed overall size (n=[10, 100, 1000, 10000]) were randomly populated with residues drawn in proportion to their expected frequency according to the Swiss-Prot human proteome. These sample sizes were selected based on the anticipated sample sizes of the data sets likely to be supplied by users as inputs to this tool. Variants of these data sets were then generated by substituting a variable number of either leucine or tryptophan into a single position

of the sequences. The number of substituted sequences was increased from 10% to 100% of the overall sample size in steps of 10% (1 sequence, 10 sequences, 100 sequences, or 1000 sequence, respectively). 100 replicates were generated for each combination of sample size and pattern percentage frequency, by replacing the randomly selected positional residues.

Each sample was analyzed and F1 scores were calculated to characterize the accuracy of the pattern detection algorithm across replicates. Mean F1 scores were then plotted, with results partitioned into separate figures for each overall sample size and scores corresponding to each pattern plotted on the same axis.

Results

As expected, these experiments reveal that the minimum percentage frequency of a pattern depends on the overall sample size and, by extension, on the absolute frequency of sequences corresponding to both the pattern and noise. In the analyses featuring an overall sample size of 10 sequences, detection of the pattern containing leucine reached 50% accuracy when the percentage frequency of sequences containing the pattern was between 60% and 70%. This is equivalent to 6 or 7 sequences given the sample size and represents a rightward shift of absolute frequency required for detection shown in Figure S5. Moreover, non-zero F1 scores were observed for this pattern at lower percentage frequency levels, indicating that this shift is not solely due to the minimum percentage frequency necessary for the pattern to exceed the p-value threshold. It is thus clear that, particularly in very small samples, this algorithm's limits of detection are governed not only by the absolute frequency of sequences containing a particular pattern, but also by the sources of error which arise when the percentage frequency of sequences matching a particular pattern is less than 100%.

This conclusion is supported by the observation that this trend persists in analyses of samples of greater overall size. While tryptophan-defined patterns are reliably detectable at levels down to at least 10% frequency, patterns defined by leucine exhibit lower accuracy of detection at such low percentage frequencies. However, in samples of 100 sequences or more, accuracy is extremely stable and close to 100% when pattern percentage frequency exceeds the limit of detection. Additionally, the difference of this lower bound for the least and most abundant residues diminishes as sample size is increased.

Investigation of pattern frequency fold difference

Objective

The prior analyses have dealt exclusively with samples containing a single enriched pattern. However, it is likely that the majority of use cases of this algorithm will involve analysis of datasets containing multiple enriched patterns arising from more than one biological activity. It is also likely that the levels of activity of these multiple processes will differ and, therefore, that the enriched patterns arising from their specificities will vary in frequency.

When the activity of one biological activity exceeds that of another, it is possible for the enrichment arising from the first to mask that of the second when evaluated simultaneously. This algorithm addresses that issue through the implementation of a set reduction operation which isolates sequences containing the most enriched positional residue producing two subsets, one containing those sequences and the other containing the remainder of sequences, during each iteration.

The purpose of this experiment is to demonstrate that, provided a first positional residue is enriched in the context of a set of sequences, and provided that a second positional residue is enriched in the context of the subset of those sequences not containing the first positional residue, the overall frequency of the first positional residue may be increased *ad nauseum* without impacting the likelihood of detecting the second positional residue.

Methods

For each replicate, a constant number of sequences (n=20 sequences) containing a single fixed positional residue were generated, where all non-fixed positions were populated randomly based on expected amino acid frequencies calculated from the Swiss-Prot human proteome. These sequences were then concatenated with a second set of sequences containing a different fixed positional residue. The sample size of the second set of sequences was set to either 1x (20 sequences), 10x (200 sequences), 100x (2000 sequences), or 1000x (20,000 sequences) the number of sequences in the first set. 100 replicates were generated for each pattern frequency fold difference condition by substituting the non-fixed positional residues.

Each replicate was analyzed twice: once with the algorithm's set reduction operation enabled and once with set reduction disabled. Mean F1 scores were calculated and plotted on a single figure for direct comparison of results for each sample condition across the two algorithm configurations.

Results

Predictably, the algorithm's set reduction operation is crucial for both the accurate detection of multiple patterns in the same dataset, as well as the accurate rejection of spuriously over-represented positional residues. As is evident in Figure S6, with this set reduction enabled, the performance limitations discussed in these analyses apply only to the most enriched positional residue at point in an analysis. Removal of that positional residue from both the foreground and background data sets for subsequent iterations ensures that it does not interfere with detection of other enriched positional residues. This behavior ensures mean accuracy of greater than 95%, even cases of extreme fold difference between the foremost and next-most enriched patterns in a dataset.

The logic of this conclusion can be extended to datasets containing more than two patterns, provided each pattern exceeds the minimum absolute and percentage frequency thresholds with respect to either the full input set for the most over-represented pattern, or the subset of sequences excluding all patterns which are more enriched.

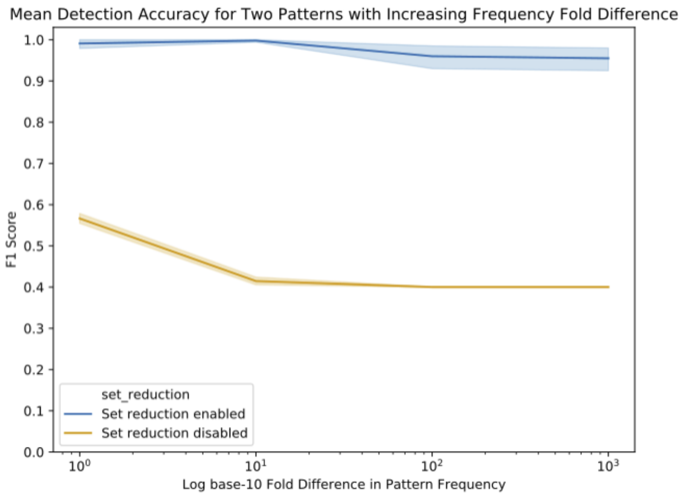

Figure S6. Mean Detection Accuracy for Two Patterns with Increasing Frequency Fold Difference. Mean F1 scores for the sequential detection of two enriched patterns with frequency fold difference increasing from 1x to 1000x in steps of 1 order of magnitude per iteration. The blue line shows scores corresponding to analysis of samples with the algorithm's set reduction operation enabled, and thus shows results when the first of the two patterns in removed from the main sequence set following its detection. The yellow line shows results with the set reduction operation disabled.

Investigation of number of detectable top-level patterns

Objective

In addition to assessing the algorithm's performance on samples containing patterns with variable frequency fold difference, it is also critical to assess the performance of this algorithm on samples containing a variable number of patterns. A key advantage of this algorithm over many alternative clustering algorithms is that the number of anticipated patterns does not need to be specified in advance. This number can be difficult to predict and often involves a process of repeated clustering in order to probe for the correct solution. While there are quantitative methods available to support this process, it remains error prone and biased. It is therefore desirable to demonstrate the reliability of this algorithm on datasets featuring a wide range of patterns and, moreover, to characterize the algorithm's capacity to resolve an unknown number of patterns accurately and exhaustively.

Methods

As demonstrated in the previous analysis, the proportional representation of two patterns does not impact the accuracy with which the algorithm detects either pattern, provided each pattern's frequency exceeds the minimum absolute and percentage frequency thresholds for detection. Therefore, to simplify the design of data sets for these experiments as well as to minimize sources of variation, frequency was held constant at 50 sequences for all patterns in this analysis, while overall sample size was held constant at 1000 sequences. This is equivalent to a percentage frequency of 5% for each pattern in the input data sets, which is at the lower end of the detectable range as discussed in the evaluation of the minimum pattern percentage frequency detectable by this tool. Each data set was populated with a variable number of patterns with the total number increasing from 1 (5% of the total sample size cumulatively) to 20 (100% of the total sample size cumulatively).

These data sets included two variants: one containing enriched positional residues in random positions and one containing enriched positional residues in the same position across patterns. While the first variant is likely to more closely approximate what would be encountered in a user-supplied data set, the second variant is intended to approximate the most challenging case for the algorithm. With all enriched residues occurring in the same position, the algorithm is likely to identify several of the enrichments as a single, aggregated compound residue enrichment. Due to the fact that biochemically similar amino acids tend to occur at similar frequencies in the proteome, and that all patterns are represented in equal frequency in these data sets, these compound residues are very unlikely to be reliably decomposed into individual amino acid enrichments by the algorithm. As each of these events produces one or more false negatives as well as one or more false positives, they dominate the errors anticipated in this analysis. Therefore, the results corresponding to these data sets represent the lower extreme of accuracy

that could be expected in real world applications. 100 replicates were produced for each combination of positional enforcement and number of patterns.

Results of the analyses were partitioned based on the positional enforcement in the set design and plotted as separate lines on the same figure. This figure displays mean F1 scores as a function of the number of patterns in the datasets with scores averaged across replicates.

Results

As depicted in Figure S7, performance was found to degrade slightly as the number of patterns increased when patterns were allowed to occur in any position, selected at random. This is close to, but slightly worse than, the expected outcome of stable performance up to the maximum number of patterns based on fixed pattern frequency. This is the result of a combination of factors including erroneous aggregation and subsequent failure of decomposition of compound residues as well as cross-talk between patterns in different positions which coincidentally feature one or more positional residues corresponding to another pattern. Given that pattern percentage frequency was selected to be close to the lower bound of detection, any depletion of pattern exemplar sequences due to set reduction has relatively high potential to lead to a false negative.

Due to the factors discussed in the previous section, performance is markedly worse when all patterns are forced to occur in the same position with mean accuracy dropping below 50% when 14 or more patterns are present. However, this is an extremely unlikely occurrence and only serves to show the extreme low end of performance. At the other end of this spectrum, a sample which only contains patterns with fixed residues in different positions would likely exhibit the lowest error rate. However, this would limit the total number of single residue patterns that could be detected to 8 based on the width of the sequences included in these data sets. On those grounds, as well as on the grounds that these analyses are intended to reveal the limitations rather than the ideal performance of this algorithm, those experiments were not conducted.

Investigation of false positive rate in randomly selected Swiss-Prot sequences

Objective

All prior analyses have dealt with samples containing one or more patterns, known to be enriched based on the construction of each input data set. However, an alternative anticipated scenario is that input samples may contain no over-represented positional residues relative to expected residue frequencies. In other words, one would expect that a biological sample collected at random from normal tissue or fluid would consist of a mixture of proteins containing amino acid frequencies that cumulatively approximate the expected background frequencies of that organism and tissue or fluid type.

It is therefore critical to evaluate the algorithm's ability to correctly reject all spuriously overrepresented positional residues in samples which should not contain any enriched patterns. Moreover, it is important to demonstrate this ability when using default algorithm settings which have already been demonstrated to be capable of detecting truly enriched patterns with high accuracy.

This analysis evaluates the rate of false positives discovered by this algorithm in samples of various sizes corresponding to the sizes selected for prior analyses as well as the sizes anticipated to be supplied as input to the algorithm by users.

Methods

Two types of samples were constructed for this analysis. Firstly, samples of 10, 100, 1,000, and 10,000 random sequences were generated by selecting positional amino acids in proportion to their expected frequencies as calculated using the Swiss-Prot human proteome. Secondly, random sub-sequences were selected directly from the protein sequences included in the Swiss-Prot human proteome. 100 replicates were produced for each sample size and sequence type combination, consisting of different randomly generated or proteome-derived sequences, respectively.

Each of these samples was then analyzed twice: once with compound residues enabled and once with compound residues disabled. Results were then partitioned into combinations of sample size, sequence type, and compound residue status and mean F1 scores were computed across all 100 replicates of each

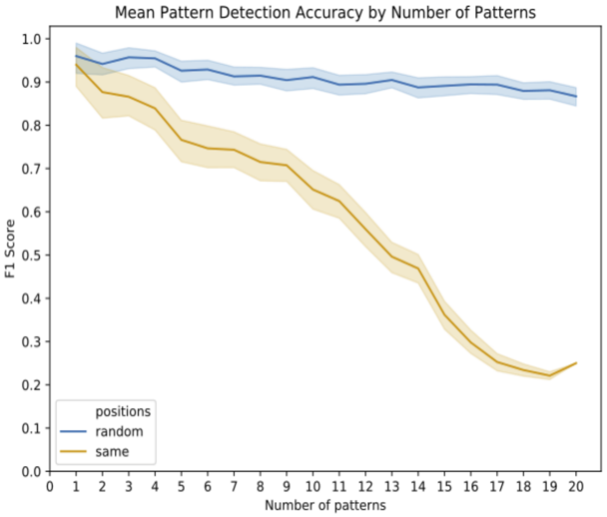

Figure S7. Mean Pattern Detection Accuracy by Number of Patterns. Mean F1 scores across replicates for samples (n=1000) containing between 1 and 20 patterns comprised of 50 sequences each. The blue line indicates mean F1 scores where the fixed position of each pattern was selected at random, while the orange line indicates mean F1 scores where all patterns exhibited the same fixed position.

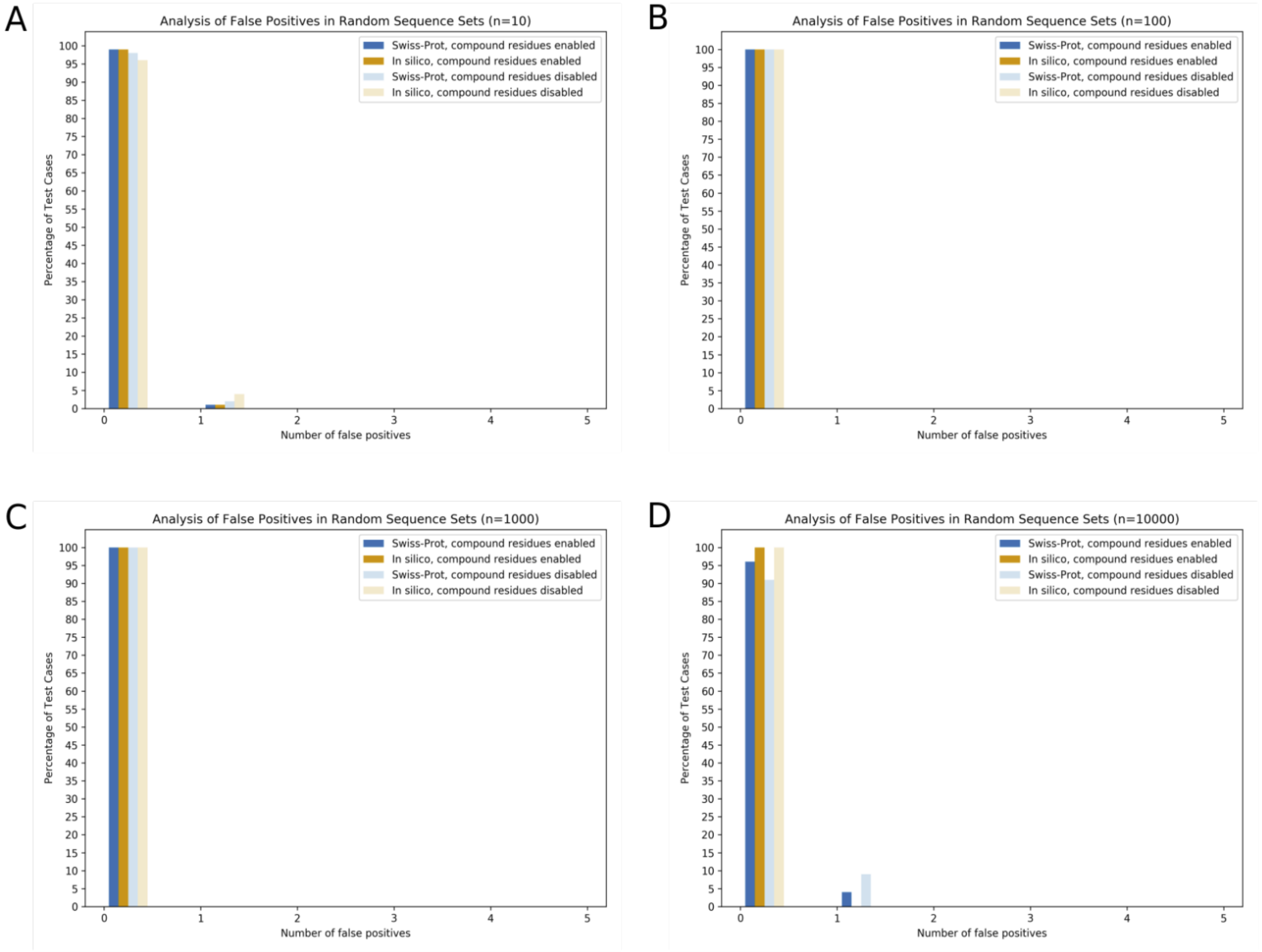

Figure S8. Analysis of False Positive Rate in Random Sequence Sets. Histograms displaying number of false positives by percentage of replicates for each combination of sample type and compound residue status. Sample types were characterized by either *in silico* sequences populated with residues selected at frequencies proportional to their expected frequency given the Swiss-Prot human proteome as a background, or sequences selected at randomly directly from the proteins comprising the Swiss-Prot human proteome.

combination. Mean F1 scores for each sample size were plotted on a separate figure, with each combination of sequence type and compound residue status plotted in a different color on the same set of axes. Histograms were generated to convey the number of false positives as a percentage of test cases.

Results

The results of this analysis, visualized in Figure S8, demonstrate the robustness of this algorithm to false positives, even in very large data sets (in terms of the anticipated use cases). Importantly, false positives are not significantly more prevalent in samples of sequences drawn directly from the human proteome as compared to samples of *in silico* sequences. This is relevant as real-world input to the algorithm will consist of sequences directly from the proteome, rather than sequences deliberately constructed to reflect the expected background frequencies of each residue.

Enabling the detection of compound residues did not increase the frequency of false positives. In the samples containing 10,000 sequences, the outcomes for analyses with compound residues enabled showed slightly better performance. While this is almost certain to be the result of chance, these analyses taken together suggest that compound residues do not hinder the ability of the algorithm to correctly reject false negatives.

Investigation of varying sequence widths on algorithm performance

Objective

Sequences analyzed in RoLiM have so far contained variable amino acid length (window width) between 8 and 15. However, it is possible that users of this algorithm will have sequence widths within 8 to 16 amino acids, as is customary for PTM motif detection.

The aim of this test is to establish that the performance of this algorithm is not affected by window width selections between the optimal 8 to 16 amino acid lengths.

Methods

To evaluate the effect of different window widths on RoLiM's performance, we generated a 16,000 amino acid long sequence matching human amino acid frequencies using the Expasy tool[6]. This sequence was split into 1000 sequences of length 16. Two patterns, frequent enough to be detected, were simulated using the 1st to 125th sequences by (i) changing the first 75 sequences to (9x)SxxSP(11x), (ii) changing the next 50 sequences to R(11x)TXXR(9x), where x is any random amino acid. All sequences were then truncated by 1, alternating from left and right end, to generate sequence sets of length 16 down to 8. To maintain pattern (ii), the first amino acid in truncated sequences was repeatedly replaced with an R. Each sequence subset was analysed in RoLiM to determine if one or both motifs described in (i) and (ii) above were correctly detected and how many other motifs were identified. The default algorithm parameters were maintained for all analyses on subsets.

Results

The results in Table S6 show that the algorithm performance consistently detected the same enriched motifs (i and ii above). This was consistent for all nine sequence subsets, irrespective of window width selected, at a pattern frequency threshold of 0.075 and 0.05 respectively.

RoLiM Analysis Output

Window Width		Pattern	Sample Frequency	Foreground Frequency	Foreground Size
16					
	0	[R]......[T]...[R]....	51	51	56
	1	.....[S].[S][P].......	75	75	81
15					
	0	[R].....[T]...[R]....	50	50	54
	1	....[S].[S][P].......	75	75	81
14					
	0	[R].....[T]...[R]...	50	50	54
	1	....[S].[S][P]......	75	75	81
13					
	0	[R]....[T]...[R]...	52	52	54
	1	...[S].[S][P]......	75	75	81
12					
	0	[R]....[T]...[R]..	52	52	54
	1	...[S].[S][P].....	75	75	81
11					
	0	[R]...[T]...[R]..	50	50	59
	1	..[S].[S][P].....	75	75	81
10					
	0	[R]...[T]...[R].	50	50	59
	1	..[S].[S][P]....	75	75	81
9					
	0	[R]..[T]...[R].	50	50	53
	1	.[S].[S][P]....	75	75	81
8					
	0	[R]..[T]...[R]	50	50	53
	1	.[S].[S][P]...	75	75	81

Table S6. Analysis of RoLiM performance using different sequence widths. Results of analysis on 9 sequence sets each consisting of 1000 sequences with window widths of 16 to 8 amino acids (as described in Methods). The same enriched Pattern consisting of 2 motifs was detected at default RoLiM settings (p-value = 0,001).

Comparison of RoLiM with Motif-X algorithm / re-implementations

To support understanding of the advanced features of RoLiM over Motif-X, rmotif-X and MoMo, a summary of the motif deconvolution approaches of these algorithms is provided in Supplementary Table S7.

	RoLiM	Original Motif-X (no longer available)	rmotif-X	MoMo
Settings and Operation				
Central residue specification required	No	Yes	Yes	No
N-terminal sequence extension and alignment	Yes	Yes	No	Yes
C-terminal sequence extension and alignement	Yes	No	No	No
Redundant sequence removal	Optional: none, protein, sequence	No	No	Optional: none, sequence
Compound residue groups	Optional: none, default, user-supplied	No	No	No
Multiple maximally-enriched position/residue pairs	Branches when encountered	Selects one, platform dependent	Selects one, platform dependent	Selects one, algorithm dependent
Background frequency calculation	Optional: position-specific (default), averaged	Position-specific	Position-specific	Optional: shuffled foreground (default), position-specific
Enhanced p-value precision	Yes	No	Optional	Optional
Positional bias	No	Not tested	Yes	No
Sequence order bias	No	Not tested	Not tested	Yes
Output data				
Visualization of motifs	Yes	Yes	No	Yes
Hierarchical sequence/motif clustering	Yes	No	No	No
Enhanced differential analysis	Yes	No	No	No

Table S7. A concise comparison of the features of RoLiM with Motif-X algorithm and its re-implementation. Different steps and approaches for motif deconvolution are utilized by each algorithm. The table compares the utility of each tool for robust and accurate identification of enriched patterns, and additional downstream processing of output data.

Additional references

1. Crooks, G. E., Hon, G., Chandonia, J. M. & Brenner, S. E. WebLogo: a sequence logo generator. *Genome Res.* **14**, 1188–1190 (2004).

2. UniProt Consortium. UniProt: a worldwide hub of protein knowledge. *Nucleic Acids Res.* **47**, D506–D515 (2019).

3. Henikoff, S. & Henikoff, J. G. Amino acid substitution matrices from protein blocks. *Proc Natl Acad Sci USA* **89**, 10915–10919 (1992).

4. Dunn, O. J. Multiple comparisons among means. *J. Am. Stat. Assoc.* **56**, 52 (1961).

5. Voorhees, E. M. Implementing agglomerative hierarchic clustering algorithms for use in document retrieval. *Information Processing & Management* **22**, 465–476 (1986).

6. Artimo, P. *et al.* ExPASy: SIB bioinformatics resource portal. *Nucleic Acids Res.* **40**, W597-603 (2012).
